# Immunogenomic correlates of immune-related adverse events for anti–programmed cell death 1 therapy

**DOI:** 10.3389/fimmu.2022.1032221

**Published:** 2022-11-25

**Authors:** Lei Zhang, Yuankai Shi, Xiaohong Han

**Affiliations:** ^1^ Department of Medical Oncology, National Cancer Center/National Clinical Research Center for Cancer/Cancer Hospital, Chinese Academy of Medical Sciences & Peking Union Medical College, Beijing Key Laboratory of Clinical Study on Anticancer Molecular Targeted Drugs, Beijing, China; ^2^ Medical Research Center, Key Laboratory of Digital Technology in Medical Diagnostics of Zhejiang Province, Hangzhou, China; ^3^ Clinical Pharmacology Research Center, Peking Union Medical College Hospital, State Key Laboratory of Complex Severe and Rare Diseases, NMPA Key Laboratory for Clinical Research and Evaluation of Drug, Beijing Key Laboratory of Clinical PK & PD Investigation for Innovative Drugs, Chinese Academy of Medical Sciences & Peking Union Medical College, Beijing, China

**Keywords:** immune-related adverse event, cellular biomarker, molecular biomarker, immune cell, immunotherapy

## Abstract

Despite impressive antitumor efficacy of programmed cell death 1 (PD-1) inhibitors, this inhibition can induce mild to severe autoimmune toxicities, termed immune-related adverse events (irAEs). Yet, predictive pretreatment biomarkers for irAEs development across cancer types remain elusive. We first assessed cellular and molecular factors. To determine factors predicting the risk of irAEs for anti–PD-1 immunotherapy across multiple cancer types, an integrative analysis of cellular and molecular factors from 9104 patients across 21 cancer types and 4865522 postmarketing adverse event reports retrieved from adverse event reporting system was then performed. Accuracy of predictions was quantified as Pearson correlation coefficient determined using leave-one-out cross-validation. Independent validation sets included small cell lung cancer and melanoma cohorts. Out of 4865522 eligible adverse events reports, 10412 cases received anti–PD-1 monotherapy, of which, 2997 (28.78%) exhibited at least one irAE. Among established immunogenomic factors, dendritic cells (DC) abundance showed the strongest correlation with irAEs risk, followed by tumor mutational burden (TMB). Further predictive accuracy was achieved by DC and TMB in combination with CD4^+^ naive T-cells abundance, and then validated in the small cell lung cancer cohort. Additionally, global screening of multiomics data identified 11 novel predictors of irAEs. Of these, *IRF4* showed the highest correlation. Best predictive performance was observed in the *IRF4* – *TCL1A* – SHC-pY317 trivariate model. Associations of *IRF4* and *TCL1A* expression with irAEs development were verified in the melanoma cohort receiving immune checkpoint inhibitors. Collectively, pretreatment cellular and molecular irAEs-associated features as well as their combinations are identified regardless of cancer types. These findings may deepen our knowledge of irAEs pathogenesis and, ultimately, aid in early detection of high-risk patients and management of irAEs.

## Introduction

Immune checkpoint inhibitors (ICIs) targeting programmed cell death 1 (PD-1) pathway have brought remarkable clinical benefits in diverse cancers (1). The ICIs work by blocking the PD-1 from binding with its partner proteins, resulting in immune activation in the tumor microenvironment (2). Nevertheless, ICI use is commonly associated with autoimmune toxicities, known as immune-related adverse events (irAEs) (3). The most common irAEs are observed in skin, colon, endocrine organs, liver, and lungs, but any organ can be affected and some infrequent irAEs may be serious and fatal, such as encephalitis and myocarditis (3, 4). Pre-existing autoantibodies (5), gut microbiome (6), tissue-resident T-cells (7), microRNAs (8), and cytokines (6) have all been involved in irAEs in single cancer type. The pathogenesis of irAEs remains poorly characterized and no biomarkers are routinely used in standard clinical practice to recognize patients at high risk for irAEs development.

Although high tumor mutational burden (TMB) has recently been reported to correlate with elevated irAEs risk across cancer types (9), large proportion (> 50%) of variation in irAEs risk has not yet been accounted for during anti–PD-1 therapy, indicating the role of other factors in leading to irAEs. Herein we systematically study this hypothesis, aiming to identify additional pretreatment immunogenomic factors that contribute to irAEs development regardless of cancer types. To this end, we analyzed cleaned large-scale pharmacovigilance data of irAEs from The US Food and Drug Administration’s Adverse Event Reporting System (FAERS) and The Cancer Genome Atlas (TCGA) multiomics data from whole-exome sequencing, mRNA sequencing, microRNA sequencing, and reverse phase protein array across multiple cancer types, and lastly, validated our hypothesis in independent cohorts.

## Materials and methods

Details about the methods are provided in **Supplementary Methods**, and a flow chart illustrating main analyses conducted is presented as **Supplementary Figure S1**.

### IrAEs risk evaluation

FAERS is a database of spontaneously gathered adverse event reports, containing great collection of reports of irAEs on anti–PD-1 agents in a real-world situation. A FAERS search engine named OpenVigil (version 2.1) was used to retrieve cleaned adverse event reports (10). Only reports with nivolumab or pembrolizumab as the suspected cause of adverse events were considered. Further, given that overall prevalence of irAEs and severity was higher with combined PD-1 and cytotoxic T-lymphocyte associated protein 4 (CTLA-4) antibodies as compared with monotherapy (11, 12), anti–PD-1 agents plus ipilimumab combination therapy was excluded. The irAEs reported in FAERS were defined as 106 preferred terms in the Medical Dictionary for Regulatory Activities according to previously published irAEs management guidelines during ICI therapy (3, 4, 13, 14), and listed in **Supplementary Table S1**. Lastly, reporting odds ratio (ROR) was calculated for each cancer type to evaluate the risk of a cancer type developing any irAE, which represents standard practice for quantitative analyses of data in spontaneous reporting systems such as FAERS (9, 15, 16).

### Molecular and cellular data sources from TCGA

Datasets of somatic mutations, mRNA, microRNA, and protein expression for 9104 samples across 21 cancer types (**Supplementary Figure S1**) with available irAEs ROR data were downloaded from the TCGA Pan–Cancer Atlas project hosted in the UCSC Xena Hubs (17). TMB was then calculated as the count of nonsynonymous mutations for each patient based on somatic mutations, and log-transformed. On the basis of the mRNA expression dataset, several tumor immune microenvironment-related signatures were generated, including cytolytic index to assess intratumoral cytolytic activity (18), interferon (IFN)-gamma and expanded immune signatures (19), and a transcriptional signature reflecting CD8^+^ T-cells exhaustion (20). Proportion of PD-1-high samples for each cancer type was also determined, with percentile 80^th^ of PD-1 mRNA expression in the entire TCGA cohort as the cutoff (21).

Other immunogenomic factors, including T cell receptor (TCR) diversity, intratumor heterogeneity, and neoantigen load, were obtained from Genomic Data Commons Pan-Cancer Atlas panimmune data portal (22).

Abundance data of 30 immune cell types in the tumor microenvironment for the TCGA samples were inferred using xCell (23) and downloaded from the xCell website. The abundance was defined as an enrichment score which showed resemblance to the fraction of specific cell type in the tumor microenvironment (23).

Lastly, median values of individual aforementioned immunogenomic factors except the PD-1-high samples proportion were calculated for each cancer type. Raw data of mRNAs, microRNAs, proteins, and phosphoproteins were preprocessed separately, and then their median expression levels per cancer type were determined for further analyses.

### Objective response rates across cancer types

Objective response rate (ORR) for PD-1 or its ligand PD-L1 inhibitor monotherapy across 19 types of cancers (**Supplementary Figure S1**) was compiled from Yarchoan et al. (24). To evaluate the correlation of tumor response with irAEs risk, Pearson correlation coefficient (*R*) between the ORR and the corresponding irAEs ROR across these cancer types was calculated.

### Small cell lung cancer and melanoma cohorts

Given that molecular data for small cell lung cancer is lacking in TCGA but its irAEs ROR could be calculated in our study, we focused on an independent cohort encompassing 71 small cell lung cancer patients with both somatic mutations and mRNA sequencing data of pretreatment tumors available (25). TMB was calculated as previously described. To estimate abundances of immune cells, gene expression data in fragments per kilobase of exon per million reads mapped units was fed to the R package xCell (23). The ICIs-treated cohort in our study consisted of 60 patients with metastatic melanoma, which received either anti–PD-1 blockade (nivolumab or pembrolizumab) or nivolumab plus ipilimumab therapy (26). All irAEs were classified according to the United States Health and Human Services Common Terminology Criteria for Adverse Events v.5.0. Grade 0 reflected no toxicity, and irAEs occurrence was defined as grade 1+. RNA sequencing was performed for peripheral blood mononuclear cell samples obtained at baseline, and then read counts were normalized to gene-level transcripts per million (TPM) for further differential gene expression analyses against irAEs status.

### Statistical analysis

To examine the relationships of molecular and cellular factors with irAEs risk, Pearson correlation analysis was used to calculate the *R*s between their respective medians and the ROR across the 21 cancer types above. For combinations of irAEs risk-associated factors, a multivariable linear regression analysis with leave-one-out cross-validation in predicting ROR across cancer types was performed using the R package caret. Prediction performance of linear models was determined as *R* and unexplained variance (1 − R^2^). Multicollinearity among variables in a multivariable linear model was quantified as variance inflation factor (VIF) calculated using the R package rms; a VIF > 4 was considered as an indicator of multicollinearity. Log-likelihood ratio test was applied to comparing the goodness-of-fit between different models using the R package lmtest. Specifically, the log-likelihood ratio test was conducted between the bivariate model and corresponding single variable models to determine the bivariate model fitness; for the trivariate model fitness comparison, the log-likelihood ratio test was conducted between the trivariate model and corresponding bivariate models. Multiple testing correction was performed to control the false discovery rate (FDR) by the Benjamini-Hochberg method. All *P* values were 2-sided and statistical significance was expected at FDR <.05 unless stated otherwise.

In the melanoma cohort, Mann-Whitney *U* test was used to compare the difference in gene expression between irAEs status. To eliminate the possibility that the associaton between gene expression and irAEs status was skewed by ICIs therapy type, a logistic regression model was adopted to control for different therapy classes. All statistical analyses were done in R statistical software v.3.5.2.

## Results

### Association of established immunogenomic factors with irAEs risk

A total of 4865522 reports were identified in FAERS, including 10412 cases that received the anti–PD-1 monotherapy for 22 cancer types. Of those 10412, 2997 cases (28.78%) exhibited at least one irAE. As shown in **Supplementary Figure S2**, the irAEs RORs varied between cancer types, ranging from the lowest 0.94 in cholangiocarcinoma to the highest 5.87 in melanoma.

**Figure 1:**
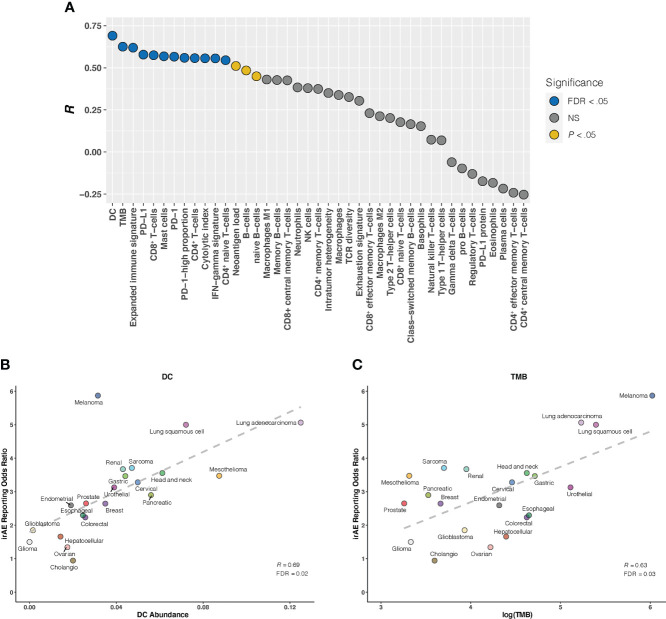
Correlation Between Established Immunogenomic Factors and Immune-Related Adverse Events for Anti–PD-1 Therapy Across Cancer Types **(A)**, Evaluation of immunogenomic correlates of immune-related adverse events (irAEs) for immunotherapy to block the PD-1 pathway across cancer types. The horizontal axis denotes the 41 established immunogenomic factors, and the vertical axis denotes Pearson correlation coefficient (*R*). Circles are filled with distinct colors as per statistical significance of corresponding factors. **(B, C)**, Correlation of dendritic cells abundance (DC) **(B)** and log(tumor mutational burden [TMB]) **(C)** with the reporting odds ratio of any irAE across 21 cancer types which are color coded. The dashed line depicts the linear fit. FDR, false discovery rate; NS, not significant.

Given that the relationship between irAEs onset and survival advantage of patients on ICIs has been shown in large case series studies in multiple cancers (27), we first examined the correlation of irAEs ROR with ORR. Our analysis demonstrated a significant positive correlation between them (*R* = 0.51; *P* = .03) (**Supplementary Figure S3**). Next, we investigated whether established immunogenomic correlates of response to ICI therapy may associate with irAEs risk. Strong association signals were identified for 15 factors (*P* <.05 for all), with 12 passing the correction for multiple testing (FDR <.05 for all): 5 immune cells, TMB, 3 immune expression signatures, and 3 checkpoint-related factors. (**Figure 1A**). Specifically, the strongest correlation between dendritic cells (DC) abundance and ROR was observed (*R* = 0.69; FDR = .02) (**Figure 1B**), suggesting that 48% of the differences in ROR across cancer types can be explained by DC. Estimated abundances of all other immune cell types were not significantly correlated with irAEs risk, except for CD8^+^ T-cells (*R* = 0.57; FDR = .04), Mast cells (*R* = 0.57; FDR = .04), CD4^+^ T-cells (*R* = 0.56; FDR = .04), and CD4^+^ naive T-cells (*R* = 0.55; FDR = .04) (**Figure 1A**, **Supplementary Figures S4A–D**). Consistent with the previous study (9), elevated TMB was demonstrated to correlate with increased risk of irAEs (*R* = 0.63; FDR = .04) (**Figure 1C**). Additionally, 3 immune expression signatures — expanded immune signature, IFN-gamma signature, and cytolytic index — which are related to IFN-gamma signaling and activated T cell biology (18, 19), displayed significant correlations with ROR (**Supplementary Figures S4E–G**). We also found that checkpoint-related factors, including individual transcriptional expressions of PD-L1 and PD-1, and PD-1-high-proportion, may contribute to irAEs onset (**Supplementary Figures S4H–J**).

### Combination of DC, TMB, and CD4^+^ naive T-cells for irAEs risk prediction

**Figure 2:**
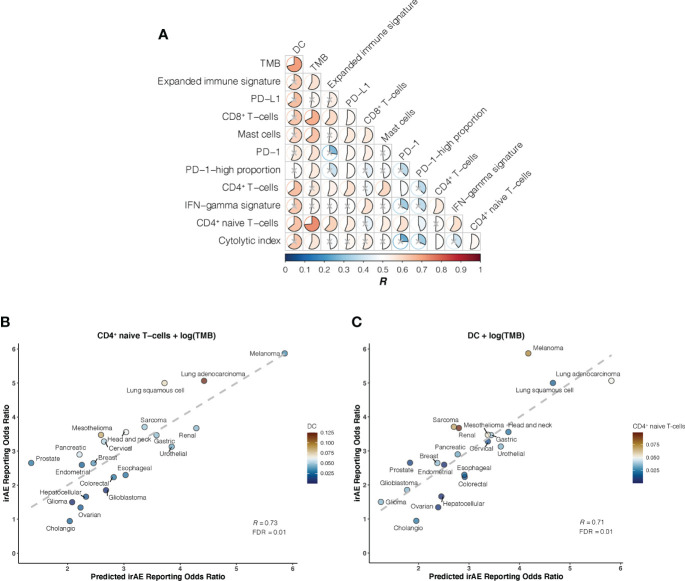
Bivariate Models of Candidate Immunogenomic Factors for Predicting Immune-Related Adverse Events for Anti–PD-1 Therapy Across Cancer Types **(A)**, Graph shows performance of bivariate models in predicting immune-related adverse events (irAEs) risk for combinations of the 12 candidate immunogenomic factors. Pearson correlation coefficient (*R*) is represented in colors from dark blue to dark red as shown in the color bar. Color intensity and the size of each pie is proportional to the correlation coefficient. A lack of statistical significance by log-likelihood ratio test (*P* >.05) is indicated with a gray cross. **(B)**, Combined effect of CD4^+^ naive T-cells abundance (naiveCD4T) and tumor mutational burden (TMB) bivariate model. The dashed line depicts the linear fit, with the formula reporting odds ratio (ROR) = 24.41 × naiveCD4T + 1.01 × log(TMB) – 2.09. The dendritic cells (DC) abundance of each cancer type is color coded where blue indicates low abundance and red, high abundance. **(C)**, Combined effect of DC and TMB bivariate model. The dashed line depicts the linear fit, with the formula ROR = 24.37 × DC + 0.8 × log(TMB) – 1.41. The CD4^+^ naive T-cells abundance of each cancer type is color coded where blue indicates low abundance and red, high abundance.

**Figure 3:**
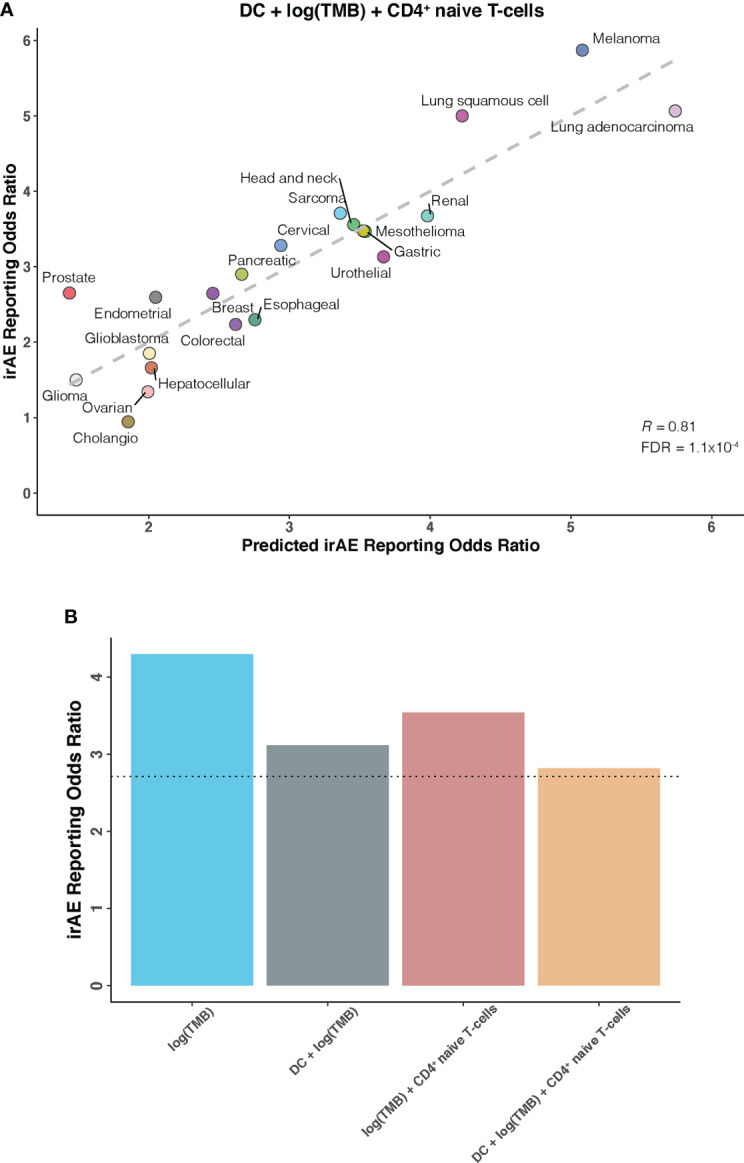
DC – TMB – CD4^+^ Naive T-cells Trivariate Model for Predicting Immune-Related Adverse Events for Anti–PD-1 Therapy Across Cancer Types **(A)**, Combined effect of dendritic cells abundance (DC) – tumor mutational burden (TMB) – CD4^+^ naive T-cells abundance (naiveCD4T) trivariate model. The dashed line depicts the linear fit, with the formula reporting odds ratio (ROR) = 19.03 × DC + 0.82 × log(TMB) + 18.03 × naiveCD4T – 1.85. **(B)**, Estimated ROR to anti-PD-1 therapy in small cell lung cancer based on TMB univariate model, DC – TMB and TMB – CD4^+^ naive T-cells bivariate models, and DC – TMB – CD4^+^ naive T-cells trivariate model. The dotted line represents the ROR in small cell lung cancer, which was obtained using The US Food and Drug Administration’s Adverse Event Reporting System.

We further investigated whether certain combinations of those 12 correlates of irAEs ROR could provide additional accuracy in predicting irAEs risk. The performances of 66 bivariate models were first evaluated. Of these 31 models showed significant statistical differences compared with their respective univariate models in terms of the fitness (log-likelihood ratio test, *P* <.05 for all) and no signs of collinearity were detected (VIF < 4 for all) (**Figure 2A**; **Supplementary Table S2**). However, only TMB and CD4^+^ naive T-cells or DC bivariate models outperformed the DC-based univariate model (TMB – CD4^+^ naive T-cells model, *R* = 0.73; TMB – DC model, *R* = 0.71; both FDR = .01) (**Figures 2B, C**). Of note, some cancer types, which had RORs higher than would be predicted by the TMB – CD4^+^ naive T-cells model, exhibited higher DC abundance (e.g., lung adenocarcinoma), and some with lower-than-predicted RORs showed lower DC abundance (e.g., glioma) (**Figure 2B**). The same was true for CD4^+^ naive T-cells abundance in the TMB – DC bivariate model (**Figure 2C**). Thus, we next examined whether inclusion of the third variable would aid in contributing additional information beyond the bivariate model. Indeed, of the resulting trivariate models, combined DC, TMB, and CD4^+^ naive T-cells model achieved the best predictive accuracy (*R* = 0.81; FDR = 1.1×10^-4^), and exhibited pronounced model promotion in comparison with their corresponding bivariate models (log-likelihood ratio test, *P* = 8.7×10^-4^ relative to TMB – DC model; *P* = 2.8×10^-4^ relative to TMB – CD4^+^ naive T-cells model) (**Figure 3**; **Supplementary Table S3**). Likewise, there was no sign of collinearity for this trivariate model (**Supplementary Table S3**). Collectively, these results emphasized the importance of integrating multiple factors in determining irAEs risk.

### External validation of DC – TMB – CD4^+^ naive T-cells model

Having identified candidate composite models of irAEs risk, we next attempted to verify our findings in an independent cohort of small cell lung cancer, a cancer type not depicted in TCGA and known to have high TMB but low response rates to ICIs. As shown in **Figure 3B**, estimated ROR by univariate TMB model markedly deviated from the actual ROR of 2.71. This striking deviation was also observed in previous work showing substantially lower-than-anticipated ROR for small cell lung cancer (9). However, strong improvements were seen after incorporating DC and/or CD4^+^ naive T-cells into our prediction models, further supporting the validity of synergistic combination of DC, TMB, and CD4^+^ naive T-cells.

### Dissection of novel molecular predictors for irAEs risk

**Figure 4:**
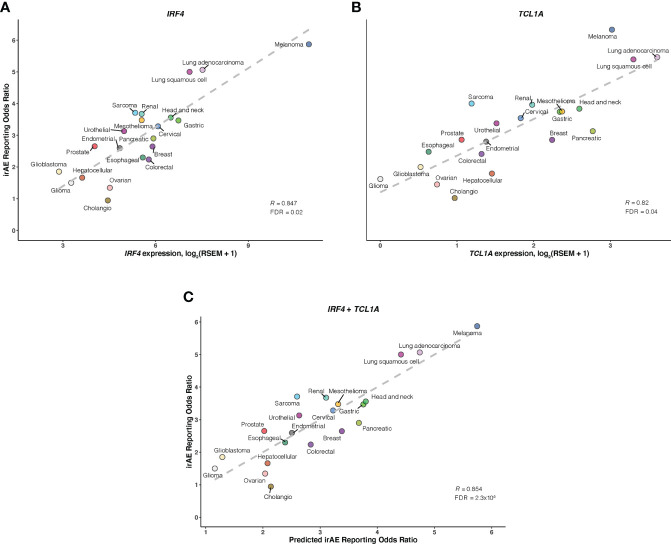
Association of *IRF4* and *TCL1A* with Immune-Related Adverse Events for Anti–PD-1 Therapy Across Cancer Types A and B, Correlation of *IRF4* **(A)** and *TCL1A* **(B)** mRNA expression with the reporting odds ratio (ROR) of any immune-related adverse event (irAE) across 21 cancer types which are color coded. The dashed line depicts the linear fit. **(C)**, Combined effect of *IRF4* and *TCL1A* expression bivariate model. The dashed line depicts linear fit, with the formula ROR = 0.38 × IRF4 + 0.54 × TCL1A – 0.1.

As a further step toward understanding irAEs development and identifying novel molecular determinants not reported to be implicated in ICI response, thus boosting irAEs risk prediction, we correlated large-scale expression profiling data for mRNA, microRNA, and protein with irAEs ROR across 21 cancer types. 11 significant predictors of irAEs risk were identified (**Figures 4A, B**; **Supplementary Figures S5-7**), such as mRNA expressions of *IRF4* (OMIM 601900), *TCL1A* (OMIM 186960), *GPNMB* (OMIM 604368), and *FAIM3* (OMIM 606015). Of these, the transcription factor *IRF4* showed the highest correlation with ROR (*R* = 0.847; FDR = .02) (**Figure 4A**), possibly relating to its essential roles in many aspects of B-cells, T-cells, and DC differentiation and function (28–31). The next highest correlation was observed for *TCL1A* (*R* = 0.82; FDR = .04) (**Figure 4B**), which is a critical player in several lymphoid malignances, and has been demonstrated to act as a coactivator to augment the activity of AKT kinases (32), thus serving as a downstream effector of B-cell receptor and TCR-mediated signaling (33). Interestingly, two additional genes, *GPNMB* (also known as *DC-HIL*) and *FAIM3*, showing positive associations with ROR (*R* = 0.81; FDR = .049) (**Supplementary Figure S5**), also had well-described roles in regulating immunity (34–37). We also noted that SHC phosphorylation level on Tyr317 (SHC-pY317) was negatively correlated with ROR (*R* = -0.75; FDR = .02) (**Supplementary Figure S6**). A study in mice indeed identified that deficiency of p66Shc protein, a homolog of human gene *SHC*, resulted in negative regulation of lymphocyte activation and autoimmunity (38). Other hits included 6 positively associated microRNAs such as miR-155-3p (*R* = 0.73; FDR = .02) (**Supplementary Figure S7**). Strikingly, miR-155 has emerged as a multifaceted mediator of innate and adaptive immunity and may drive, when deregulated, aberrant immune responses, such as the development of autoimmune disorders (39, 40).

**Figure 5:**
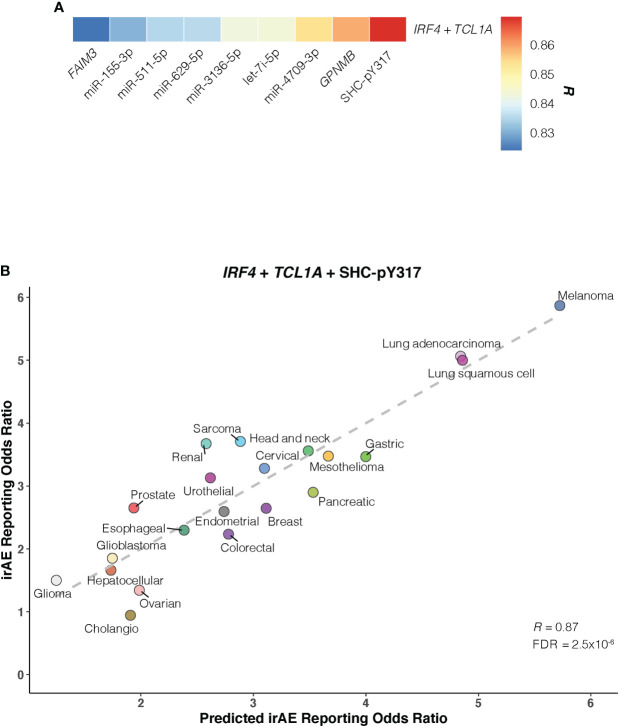
Optimal *IRF4* – *TCL1A* – SHC-pY317 Model in Predicting Immune-Related Adverse Events for Anti–PD-1 Therapy Across Cancer Types. **(A)**, Comparison of predictive performance given by the *IRF4* and *TCL1A* genes together with other immune-related adverse events (irAEs)-associated molecular factors. Pearson correlation coefficient (*R*) is represented in colors from blue to red as shown in the color bar. **(B)**, Combined effect of *IRF4* – *TCL1A* – SHC-pY317 trivariate model. The dashed line depicts the linear fit, with the formula reporting odds ratio = 0.28 × IRF4 + 0.47 × TCL1A – 2.39 × SHC-pY317 + 0.77. Cancer types are depicted in distinct colors.

Of bivariate models derived from aforementioned correlates (**Supplementary Figure S8**; **Table S4**), the *IRF4* – *TCL1A* model yielded optimal prediction accuracy (*R* = 0.854; FDR = 2.3×10^-5^) (**Figure 4C**). Although the increment of *R* was small compared with that from *IRF4* alone, the log-likelihood ratio test indicated a significant model improvement (*P* = .02 relative to *IRF4* model; *P* = 3×10^-3^ relative to *TCL1A* model). We then incorporated each of 9 other factors into the *IRF4* – *TCL1A* bivariate model successively, and found significant enhancement on the prediction performance only in the *IRF4* – *TCL1A* – SHC-pY317 trivariate vs *IRF4* – *TCL1A* bivariate models (*R* = 0.87; FDR = 2.5×10^-6^; log-likelihood ratio test, *P* = .03) (**Figures 5A, B**; **Supplementary Table S5**).

Given these results, we asked whether a composite model integrating the 11 novel molecular determinants with the 12 prior immunogenomic ones could outperform the *IRF4* – *TCL1A* – SHC-pY317 model. In contrast, none of constructed bivariate models outperformed it (**Supplementary Table S6**). Moreover, the combination of DC, TMB, CD4^+^ naive T-cells, *IRF4*, *TCL1A*, and SHC-pY317 did not improve our ability to predict irAEs risk (*R* = 0.81).

### Validation of IRF4 and TCL1A associated with irAEs in ICI-treated melanoma cohort

**Figure 6:**
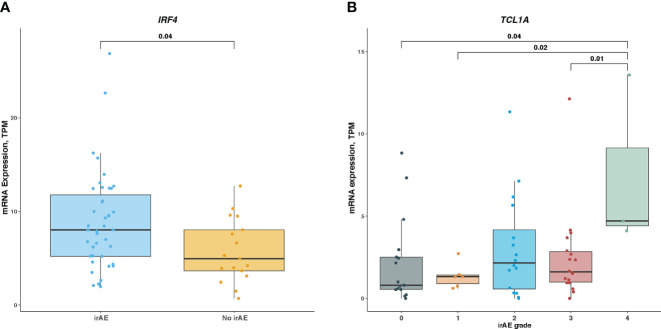
Association of *IRF4* and *TCL1A* Expression With Immune-Related Adverse Events in ICI-Treated Melanoma Cohort **(A)**, Comparison of *IRF4* mRNA level in irAEs-experiencing patients vs irAEs-free ones. **(B)**, Comparison of *TCL1A* mRNA level among different irAEs grades. Box plots show the median, first, and third quartiles; error bars extend to 1.5 times the interquartile range; and the dots denote patients profiled by mRNA sequencing. The Mann-Whitney *U* test was used to determine significance. TPM, transcripts per million.

Lastly, we examined associations of *IRF4* and *TCL1A* genes with irAEs development in patients with melanoma receiving ICI treatment. As shown in **Figure 6A**, *IRF4* mRNA level was significantly elevated in patients developing irAEs compared with those without any irAEs (median expression, 4.36 vs 3.98; Mann-Whitney *U* test, *P* = .04). We next wondered whether this association was skewed by ICI therapy type. After correcting for therapy classes, *IRF4* remained associated with irAEs development (logistic regression, *P* = .03). In contrast, the difference in *TCL1A* mRNA level stratified by irAEs status was not statistically significant, although there was a trend toward high *TCL1A* expression in irAEs-experiencing patients subgroup (median expression was 1.32 for irAEs-experiencing patients vs 0.75 for irAEs-free ones; Mann-Whitney *U* test, *P* >.05). Further analysis revealed that patients with grade 3, 1, or no irAEs had higher *TCL1A* expression than those experiencing the most severe irAEs (median expressions for grade 4, 3, 1, and 0 were 4.7, 1.61, 1.32, and 0.81, respectively; Mann-Whitney *U* test, *P* <.05 for all) (**Figure 6B**).

## Discussion

Our integrative analyses of large-scale cleaned pharmacovigilance data and multiomics profile offer a valuable collection of baseline predictors for irAEs development regardless of caner types, as illustrated by the identified 22 other risk factors beyond TMB. Moreover, proper factor combination can markedly improve the accuracy of irAEs risk prediction, emphasizing the necessity of concurrent consideration of multiple features in assessing irAEs development. Many features identified herein have been implicated in autoimmunity, thus raising the possibility of common immunological mechanisms underlying both irAEs development and autoimmune diseases.

In our work, we first investigated the relationships between the 41 established immunogenomic factors and irAEs risk, then found 12 irAEs-related factros, including 5 immune cells, TMB, 3 immune expression signatures, and 3 checkpoint-related factors. Of these, 4 cell types (DC, Mast cells, CD4^+^ T-cells, and CD4^+^ naive T-cells) have not yet been demonstrated to be associated with ICI efficacy. Next, we investigated the relationships between genome-wide mRNA, microRNA, and protein profiles and irAEs risk, and found 11 *de novo* generated irAEs-related molecular factors, all of which were not reported to be implicated in ICI efficacy. Actually, there was only moderate correlation between irAEs risk and ICI efficacy, implying that immunological mechanisms underlying irAEs development and efficacy were not completely shared. Thus we indeed identified some factors predictive of irAEs risk but not efficacy, although further experimental study is warranted to classify the biological significance of novel features identified in our study.

Based on aforementioned predictors, a trivariate model combining DC, TMB, and CD4^+^ naive T-cells, which considerably reduced the unexplained variance in predicting irAEs risk from 0.60 (1 – 0.63^2^) utilizing TMB alone to 0.34 (1 – 0.81^2^), was generated, suggesting that all these factors may get involved in the mediation of irAEs development.

We hypothesized that high TMB may contribute to increased irAEs risk due to consequent increment in immunogenic neoantigens, which could resemble peptides in normal tissues and be recognized as non-self antigens by the immune system (41), thus eliciting irAEs in target tissues as cross-reactive immune responses in the ICI therapy setting. This hypothesis invokes the theory of molecular mimicry that has been involved in the pathogenesis of autoimmune diseases, and where antibodies or TCRs recognizing pathogenic antigens could also cross-react against self-antigens (42). Evidences supporting the validity of neoantigenic molecular mimicry in the onset of irAEs come from observations in the cancer context that (1) TCRs reactive to certain neoantigens exhibited cross-reactivity to the wild-type peptides (43), and (2) shared T-cell clones were identified between tumors and target tissues of irAEs from ICI-treated patients in whom irAEs developed (44, 45).

Nonetheless, as suggested in our prediction model, it was not sufficient for immunogenic neoantigens alone to trigger irAEs, but abundant DC were required. As the most potent antigen-presenting cell type, DC are critical for priming naive T-cells by presenting antigens *via* major histocompatibility complex molecules and providing costimulatory signal (46). The engagement of DC in triggering autoimmunity has been documented *via* various mechanisms (47). For instance, deficient apoptosis of DC may increase DC numbers and lead to the onset of systemic autoimmunity (48). Additionally, previous studies suggest that DC may transfer the majority of tumor antigens from tumors to draining lymph nodes for the purpose of efficient priming of T-cells (49–51). Thus, a possible mechanism whereby neoantigenic mimicry may be implemented is that, intratumoral DC locally capture immunogenic neoantigens in tumor microenvironment, and subsequently migrate to draining lymph nodes where they disseminate neoantigens and stimulate resident T-cells. Upon being educated by DC, these T-cells would circulate systemically to induce neoantigen-specific immunopathologies such as irAEs against the cross-reactive self-antigen at distal sites.

Given the similarity between the irAEs and that of a chronic graft‐versus‐host‐disease (GVHD) reaction following allogenic hematopoietic stem cell transplantation, there is a new theory for ICIs-induced irAEs (52). It was hypothesized that ICIs induced a graft-versus-malignancy effect, which eradicated metastatic cancer in a minority of patients, but also involved an auto-GVHD reaction that leaded to widespread autoimmunity in the majority. Based on this theory, an off-label low-dose nivolumab plus ipilimumab regimen was developed and tested in 131 unselected stage IV cancer patients (53). The irAEs profile of this combined low-dose treatment was significantly safer than that of the established protocols without compromising efficacy. Our finding that DC abundance showed the strongest correlation with irAEs risk supports the auto-GVHD reaction theory as host-derived DC are also important to elicit allogeneic T cell responses (54).

Our model also highlights the potential role of CD4^+^ naive T-cells in tumor microenvironment in promoting irAEs developement. The recruitment of CD4^+^ naive T-cells into non-lymphoid tissues, including tumors, has been reported (55, 56). although their biological significances remain uncertain. It is notable that CD4^+^ T-cells are of fundamental importance in mediating autoimmunity. And this role is achieved *via* the differentiation of CD4^+^ naive T-cells into various lineages of T helper cells, depending on external cytokine microenvironment and transcription factors they induce (57).

Further performance enhancement (unexplained variance = 0.24) was seen in the composite model comprising 3 novel molecular predictors (*IRF4*, *TCL1A*, and SHC-pY317). All these features have been implicated in immune regulation (28–33, 38). Importantly, we observed elevated expression level of IRF4 in ICI-treated melanoma patients with irAEs. IRF4 is a member of the interferon regulatory factor family of transcription factors and selectively expressed in lymphoid and myeloid cells. IRF4 deletion in mice may induce transplant acceptance by establishing CD4^+^ T-cells dysfunction (58) and render mice resistant to several autoimmune diseases (28), such as ulcerative colitis and experimental autoimmune encephalomyelitis. Intriguingly, a MEK1/2 inhibitor trametinib was capable of inhibiting IRF4 expression in activated CD4^+^ T-cells (58). Collectively, these evidences suggest the therapeutic potential of targeting IRF4 expression for abrogating inflammatory toxicities from immune checkpoint blockade.

MicroRNAs are critical posttranscriptional regulators of target genes expression, and the number of microRNAs implicated in immune disorders like autoimmunity has increased dramatically (40). A recent study has shown that microRNA-146a may regulate irAEs by ICIs in mice (8). Of note, we found 6 microRNAs predictive of irAEs risk. Given that miRNAs act by targeting multiple genes within a pathway, thus causing a broader yet specific response (59), our finding may further spark the possibility of using microRNAs as therapeutics for irAEs with multifactorial origin.

We also noted a study profiled for serum cytokines/chemokines in 47 cancer patients with ICIs treatment (60). It revealed that patients with irAEs had lower baseline levels and higher posttreatment elevation in serum IFN-gamma-inducible small cytokines (CXCL9 and CXCL10). In our work, the IFN-gamma signature in tumor microenvironment showed positive correlation with irAEs risk. This observation may be associated with the difference between circulating and tumor immune microenvironment, and deserve further investigation.

This study has several limitations. First, FAERS is a spontaneous reporting database which may include reporting bias and inaccurate reports, although it has previously been used to determine risk factors linked to the development of irAEs (9, 61). Second, cancer patients with more responsive tumor immune microenvironment may remain on ICI treatment longer. However, the majority of irAEs reported during anti–PD-1 therapy occur within the first few months of commencing treatment (62), which implys that treatment duration may not bias our results. Given the identification of markers (e.g., TMB, immune signatures, and PD-L1 expression) predictive of both ICI response and irAEs risk in our study, we propose that the association between response and irAEs risk may be partially linked *via* high tumor immunogenicity and immunoresponsive microenvironment represented by these predictors. Therefore, it is necessary to discern markers able to discriminate anti–tumor efficacy from the risk of irAEs in patients with ICI treatment. Notably, all 11 novel molecular features in our study have not been reported to be associated with anti–tumor efficacy. Third, in addition to cancer-associated immunogenomic features reported in our study, host features, such as age, genetic susceptibility to autoimmunity, pre-existing autoimmune disease, and gut microbiome, may influence the development of irAEs (6). Fourth, further experimental study is required to classify the biological significance of novel features identified in our study.

In conclusion, our approach allowed us to identify cellular and molecular candidates as well as their optimal combinations for identifying patients with the risk of irAEs development during anti–PD-1 therapy, irrespective of cancer types. These findings may advance our understanding of mechanisms that drive irAEs development and tailoring personalized surveillance strategies.

## Data availability statement

The original contributions presented in the study are included in the article/**Supplementary Material**. Further inquiries can be directed to the corresponding authors.

## Ethics statement

Ethical review and approval was not required for the study on human participants in accordance with the local legislation and institutional requirements. Written informed consent for participation was not required for this study in accordance with the national legislation and the institutional requirements.

## Author contributions

YS, XH, and LZ conceived the concept and designed the study. LZ conducted statistical analysis. LZ drafted the manuscript. YS and XH performed the critical revision of the manuscript for important intellectual content. YS and XH obtained funding and supervised the study. All authors contributed to the article and approved the submitted version.

## Funding

This work was supported by China National Major Project for New Drug Innovation (2017ZX09304015, and 2019ZX09201-002).

## Conflict of interest

The authors declare that the research was conducted in the absence of any commercial or financial relationships that could be construed as a potential conflict of interest.

## Publisher’s note

All claims expressed in this article are solely those of the authors and do not necessarily represent those of their affiliated organizations, or those of the publisher, the editors and the reviewers. Any product that may be evaluated in this article, or claim that may be made by its manufacturer, is not guaranteed or endorsed by the publisher.
