# Supplementary material for: Immunogenomic correlates of immune-related adverse events for anti–programmed cell death 1 therapy
Source: Front Immunol. 2022 Nov 25;13:1032221. doi: 10.3389/fimmu.2022.1032221 (PMC9733471; doi:10.3389/fimmu.2022.1032221)
Supplement: Supplementary file 1 [file DataSheet_1.docx]

Supplementary Material

**Supplementary Methods.** IrAEs and Multiomics Data Collection

**Supplementary Table S1.** Summary of the 106 Preferred Terms to Define Immune-Related Adverse Events

**Supplementary Table S2.** Evaluation of Bivariate Models for 12 Immune-Related Adverse Events-Associated Immunogenomic Factors

**Supplementary Table S3.** Evaluation of Trivariate Models for 12 Immune-Related Adverse Events-Associated Immunogenomic Factors

**Supplementary Table S4.** Evaluation of Bivariate Models for 11 Immune-Related Adverse Events-Associated Novel Molecular Factors

**Supplementary Table S5.** Evaluation of Trivariate Models for *IRF4* and *TCL1A* Together With Nine Other Immune-Related Adverse Events-Associated Molecular Factors

**Supplementary Table S6.** Evaluation of Combination of Immune-Related Adverse Events-Associated Molecular and Immunogenomic Factors

**Supplementary Figure S1.** Flow Chart Illustrating Main analyses

**Supplementary Figure S2.** Reporting Odds Ratio of Immune-Related Adverse Event for Anti-PD-1 Therapy Across Cancer Types

**Supplementary Figure S3.** Correlation of Immune-Related Adverse Event Reporting Odds Ratio with Objective Response Rate During Anti-PD-1 Therapy Across Cancer Types

**Supplementary Figure S4.** Immunogenomic Correlates of Immune-Related Adverse Event Reporting Odds Ratio During Anti-PD-1 Therapy Across Cancer Types

**Supplementary Figure S5.** Genes Correlated With Immune-Related Adverse Event Reporting Odds Ratio During Anti-PD-1 Therapy Across Cancer Types

**Supplementary Figure S6.** Protein Correlated With Immune-Related Adverse Event Reporting Odds Ratio During Anti-PD-1 Therapy Across Cancer Types

**Supplementary Figure S7.** MicroRNAs Correlated With Immune-Related Adverse Event Reporting Odds Ratio During Anti-PD-1 Therapy Across Cancer Types

**Supplementary Figure S8.** Performance of Bivariate Models in Predicting Immune-Related Adverse Events Risk for Combinations of Candidate Genes, Protein, and MicroRNAs

**Supplementary Methods.**

***IrAEs Data***

As the practical usage of raw pharmacovigilance data (e.g., data from The US Food and Drug Administration’s Adverse Event Reporting System), is limited by several factors, such as wrong or missing data, and duplicate or multiplicate records in the database, we retrieved cleaned adverse event reports using OpenVigil (version 2.1), a open tool for data-mining and analysis of pharmacovigilance data (1). For the reliablity of immune-related adverse events (irAEs) risk evaluation, only cancer types with at least 45 adverse event reports with nivolumab or pembrolizumab as suspected causes were considered. To evaluate the risk of a cancer type developing any irAE, reporting odds ratio (ROR) was calculated as described elsewhere (2) using

$$ROR=\frac{DE \times de}{dE \times De}$$

Where: DE is the number of reports where nivolumab or pembrolizumab is used and irAEs occur, de is the number of reports where neither the agent above is used nor irAEs occur, dE is the number of reports where the agent above is not used but irAEs occur, De is the number of reports where the agent above is used but irAEs do not occur.

***Molecular Data Sources***

All datasets were downloaded from The Cancer Genome Atlas (TCGA) Pan-Cancer Atlas project hosted in the UCSC Xena Hubs (3). Exome sequencing-based mutations were uniformly called by an ensemble of seven mutation-calling tools to correct for batch effects or caller-specific biases in TCGA data (4), and TMB was then calculated as the count of somatic nonsynonymous mutations using maftools (5). Nonsynonymous mutations were defined as missense, nonsense, nonstop, frameshift insertion and deletion, in-frame insertion and deletion, and splice site mutations. The mRNA expression levels were quantified using RNA-Seq by Expectation-Maximization algorithm, batch-corrected and normalized using the upper quartile method by Hoadley et al (6). Batch-corrected, RPM-normalized microRNA expression levels were also obtained (6). Before subsequent analyses, both expression levels of mRNA and microRNA were log_2_-transformed after adding 1 offset. Protein and phosphoproteins expression levels were generated from reverse phase protein arrays and batch-corrected applying the replicates-based normalization method (7). Lastly, the median value of each factor was calculated for each cancer type.

Based on these mRNA expression data, several factors were determined. The cytolytic index to assess intratumoral cytolytic activity was determined by taking the geometric mean of *GZMA* (OMIM 140050) and *PRF1* (OMIM 170280) transcriptional levels on the original scale (8). Interferon (IFN)-gamma and expanded immune signatures were derived from the study of Ayers et al (9), and then signature scores were calculated by averaging of transcript levels of included genes in the individual signatures. A transcriptional signature (10) reflecting CD8^+^ T-cells exhaustion was generated by subtracting mean expression levels of exhaustion-specific upregulated genes by those of exhaustion-specific downregulated genes (11). Lastly, the median value of each factor was calculated as per cancer types. For calculating the proportion of PD-1-high samples for each cancer type, percentile 80^th^ of PD-1 mRNA expression in the entire TCGA cohort was used as the cutoff to define PD-1-high vs PD-1-low status (12).

Intratumor heterogeneity were determined as the subclonal genome fraction using ABSOLUTE (13) and potential neoantigenic peptides to bind with HLA class I were predicted from single nucleotides variants using NetMHCpan (version 3.0) (14), and downloaded from Genomic Data Commons Pan-Cancer Atlas panimmune data portal (15). T cell receptor diversity was measured by Shannon entropy and also downloaded from the panimmune data portal (15).

***Cellular Data Source***

xCell is a novel gene signature-based approach that integrates the advantages of gene set enrichment with deconvolution methods (16). It was applied on the TCGA tumor samples to convert their bulk gene expression profiles to enrichment scores of 30 immune cell types. These comprise dendritic cells, CD8^+^ T-cells, mast cells, CD4^+^ naive T-cells, naive B-cells, macrophages M1, memory B-cells, neutrophils, NK cells, CD4^+^ memory T-cells, monocytes, macrophages M2, type 2 T-helper cells, class-switched memory B-cells, basophils, natural killer T-cells, type 1 T-helper cells, gamma delta T-cells, pro B-cells, regulatory T-cells, eosinophils, plasma cells, CD4^+^ effector memory T-cells, CD4^+^ central memory T-cells, macrophages, B-cells, CD4^+^ T-cells, CD8^+^ effector memory T-cells, CD8^+^ central memory T-cells, and CD8^+^ naive T-cells. The median abundance of each cell type was then calculated for each cancer type.

**Supplementary Table S1. Summary of the 106 Preferred Terms to Define Immune-Related Adverse Events**

| **Preferred terms** | | | | |
| --- | --- | --- | --- | --- |
| rash | colitis | pneumonitis | autoimmune myocarditis | autoimmune pancreatitis |
| pruritus | autoimmune colitis | acute interstitial pneumonitis | pericarditis | histiocytosis haematophagic |
| pruritus generalised | colitis ulcerative | myositis | pericarditis malignant | sjogren's syndrome |
| vitiligo | colitis ischaemic | polymyositis | uveitis | sialoadenitis |
| dermatitis | colitis microscopic | polymyalgia rheumatica | autoimmune uveitis | cytokine release syndrome |
| dermatitis acneiform | enterocolitis | nephritis | iritis | synovitis |
| rash maculo-papular | enterocolitis haemorrhagic | autoimmune nephritis | episcleritis | latent autoimmune diabetes in adults |
| rash papular | primary hypothyroidism | tubulointerstitial nephritis | arthritis | stevens-johnson syndrome |
| rash erythematous | hypothyroidism | myasthenia gravis | autoimmune arthritis | toxic epidermal necrolysis |
| rash generalised | autoimmune hypothyroidism | guillain-barre syndrome | arthritis reactive | autoimmune pancytopenia |
| rash macular | hyperthyroidism | neuropathy peripheral | polyarthritis | immune-mediated adverse reaction |
| rash morbilliform | thyroiditis | autonomic neuropathy | rheumatoid arthritis | iridocyclitis |
| rash pruritic | autoimmune thyroiditis | meningitis aseptic | seronegative arthritis | autoimmune disorder |
| rash pustular | hypophysitis | encephalitis | aplastic anaemia | noninfective encephalitis |
| rash vesicular | lymphocytic hypophysitis | myelitis transverse | autoimmune haemolytic anaemia | haemolytic uraemic syndrome |
| erythema multiforme | primary adrenal insufficiency | autoimmune neuropathy | vogt-koyanagi-harada syndrome | blepharitis |
| dermatitis bullous | type 1 diabetes mellitus | encephalitis autoimmune | pemphigoid | acquired haemophilia |
| dermatitis exfoliative | fulminant type 1 diabetes mellitus | limbic encephalitis | conjunctivitis | radiation pneumonitis |
| dermatitis exfoliative generalised | hepatitis | myelitis | leukoderma |  |
| dermatitis psoriasiform | autoimmune hepatitis | encephalomyelitis | psoriasis |  |
| autoimmune dermatitis | hepatitis acute | immune thrombocytopenic purpura | pancreatitis |  |
| diarrhoea | hepatitis fulminant | myocarditis | pancreatitis acute |  |

**Supplementary Table S2. Evaluation of Bivariate Models for 12 Immune-Related Adverse Events-Associated Immunogenomic Factors**

| **Variable 1** | **Variable 2** | ***R*** | ***P*** | **FDR** | **LRT *P* for variable 1** | **LRT *P* for variable 2** | **VIF** |
| --- | --- | --- | --- | --- | --- | --- | --- |
| DC | TMB | 0.71 | 3.5E-04 | 0.01 | 1.1E-03 | 1.9E-04 | 1.08 |
| DC | PD-L1 | 0.65 | 1.4E-03 | 0.01 | 2.0E-01 | 9.4E-03 | 1.59 |
| DC | CD4^+^ T-cells | 0.65 | 1.5E-03 | 0.01 | 1.8E-03 | 7.9E-05 | 1.03 |
| DC | CD4^+^ naive T-cells | 0.64 | 1.7E-03 | 0.01 | 2.0E-02 | 6.6E-04 | 1.11 |
| DC | Cytolytic index | 0.64 | 1.8E-03 | 0.01 | 3.2E-01 | 9.0E-03 | 1.66 |
| DC | CD8^+^ T-cells | 0.64 | 1.9E-03 | 0.01 | 9.4E-02 | 4.7E-03 | 1.36 |
| DC | IFN-gamma signature | 0.63 | 2.2E-03 | 0.01 | 2.7E-01 | 7.7E-03 | 1.58 |
| DC | Expanded immune signature | 0.63 | 2.2E-03 | 0.01 | 2.1E-01 | 2.6E-02 | 1.95 |
| DC | Mast cells | 0.61 | 3.5E-03 | 0.02 | 1.1E-01 | 4.8E-03 | 1.38 |
| DC | PD-1 | 0.56 | 8.0E-03 | 0.02 | 3.0E-01 | 1.0E-02 | 1.7 |
| DC | PD-1-high proportion | 0.49 | 2.5E-02 | 0.03 | 1.7E-01 | 5.7E-03 | 1.44 |
| TMB | CD4^+^ naive T-cells | 0.73 | 1.7E-04 | 0.01 | 5.9E-04 | 1.2E-04 | 1 |
| TMB | CD8^+^ T-cells | 0.68 | 7.5E-04 | 0.01 | 3.4E-03 | 1.1E-03 | 1.05 |
| TMB | Mast cells | 0.64 | 1.6E-03 | 0.01 | 1.5E-02 | 4.3E-03 | 1.12 |
| TMB | Expanded immune signature | 0.58 | 6.2E-03 | 0.02 | 1.8E-02 | 1.6E-02 | 1.26 |
| TMB | PD-1 | 0.58 | 6.4E-03 | 0.02 | 2.9E-02 | 7.8E-03 | 1.18 |
| TMB | CD4^+^ T-cells | 0.57 | 7.1E-03 | 0.02 | 5.1E-02 | 1.1E-02 | 1.23 |
| TMB | Cytolytic index | 0.57 | 7.5E-03 | 0.02 | 2.9E-02 | 6.4E-03 | 1.15 |
| TMB | PD-1-high proportion | 0.56 | 8.4E-03 | 0.02 | 2.1E-02 | 5.1E-03 | 1.13 |
| TMB | IFN-gamma signature | 0.53 | 1.4E-02 | 0.02 | 8.5E-02 | 1.8E-02 | 1.32 |
| TMB | PD-L1 | 0.53 | 1.4E-02 | 0.02 | 5.8E-02 | 1.9E-02 | 1.32 |
| Expanded immune signature | CD4^+^ naive T-cells | 0.61 | 3.2E-03 | 0.02 | 7.4E-03 | 1.6E-03 | 1.05 |
| Expanded immune signature | CD8^+^ T-cells | 0.6 | 4.1E-03 | 0.02 | 4.2E-02 | 1.5E-02 | 1.25 |
| Expanded immune signature | PD-L1 | 0.55 | 1.0E-02 | 0.02 | 1.6E-01 | 5.6E-02 | 1.69 |
| Expanded immune signature | CD4^+^ T-cells | 0.54 | 1.1E-02 | 0.02 | 2.2E-02 | 5.8E-03 | 1.13 |
| Expanded immune signature | Mast cells | 0.52 | 1.6E-02 | 0.03 | 1.8E-01 | 5.3E-02 | 1.7 |
| Expanded immune signature | IFN-gamma signature | 0.49 | 2.4E-02 | 0.03 | 3.5E-01 | 7.0E-02 | 14.45 |
| Expanded immune signature | Cytolytic index | 0.48 | 2.6E-02 | 0.03 | 4.1E-01 | 8.0E-02 | 12.93 |
| Expanded immune signature | PD-1-high proportion | 0.39 | 7.7E-02 | 0.09 | 2.4E-01 | 5.4E-02 | 1.77 |
| Expanded immune signature | PD-1 | 0.28 | 2.2E-01 | 0.23 | 8.9E-01 | 1.5E-01 | 5.12 |
| PD-L1 | CD4^+^ T-cells | 0.58 | 6.4E-03 | 0.02 | 3.2E-03 | 2.1E-03 | 1.02 |
| PD-L1 | Mast cells | 0.56 | 7.9E-03 | 0.02 | 3.2E-02 | 2.6E-02 | 1.2 |
| PD-L1 | CD4^+^ naive T-cells | 0.56 | 8.9E-03 | 0.02 | 1.2E-02 | 6.4E-03 | 1.06 |
| PD-L1 | Cytolytic index | 0.53 | 1.3E-02 | 0.02 | 1.1E-01 | 7.0E-02 | 1.49 |
| PD-L1 | PD-1 | 0.53 | 1.3E-02 | 0.02 | 4.8E-02 | 3.7E-02 | 1.28 |
| PD-L1 | CD8^+^ T-cells | 0.53 | 1.4E-02 | 0.02 | 4.9E-02 | 4.5E-02 | 1.31 |
| PD-L1 | PD-1-high proportion | 0.51 | 1.8E-02 | 0.03 | 2.0E-02 | 1.3E-02 | 1.12 |
| PD-L1 | IFN-gamma signature | 0.5 | 2.0E-02 | 0.03 | 1.9E-01 | 1.2E-01 | 1.81 |
| CD8^+^ T-cells | IFN-gamma signature | 0.58 | 6.2E-03 | 0.02 | 2.7E-02 | 1.8E-02 | 1.15 |
| CD8^+^ T-cells | Mast cells | 0.56 | 8.1E-03 | 0.02 | 3.3E-02 | 2.9E-02 | 1.21 |
| CD8^+^ T-cells | Cytolytic index | 0.55 | 1.0E-02 | 0.02 | 6.1E-02 | 4.1E-02 | 1.29 |
| CD8^+^ T-cells | PD-1 | 0.55 | 1.0E-02 | 0.02 | 3.6E-02 | 3.0E-02 | 1.22 |
| CD8^+^ T-cells | CD4^+^ T-cells | 0.47 | 3.2E-02 | 0.04 | 7.7E-02 | 5.3E-02 | 1.36 |
| CD8^+^ T-cells | CD4^+^ naive T-cells | 0.45 | 4.1E-02 | 0.05 | 2.1E-01 | 1.1E-01 | 1.77 |
| CD8^+^ T-cells | PD-1-high proportion | 0.44 | 4.8E-02 | 0.06 | 6.1E-02 | 4.4E-02 | 1.31 |
| Mast cells | CD4^+^ T-cells | 0.6 | 3.8E-03 | 0.02 | 1.1E-02 | 9.1E-03 | 1.07 |
| Mast cells | CD4^+^ naive T-cells | 0.55 | 9.8E-03 | 0.02 | 3.0E-02 | 1.9E-02 | 1.14 |
| Mast cells | Cytolytic index | 0.51 | 1.8E-02 | 0.03 | 8.5E-02 | 6.6E-02 | 1.4 |
| Mast cells | PD-1 | 0.49 | 2.4E-02 | 0.03 | 9.7E-02 | 9.2E-02 | 1.52 |
| Mast cells | IFN-gamma signature | 0.49 | 2.4E-02 | 0.03 | 1.5E-01 | 1.1E-01 | 1.67 |
| Mast cells | PD-1-high proportion | 0.48 | 2.9E-02 | 0.04 | 5.1E-02 | 4.2E-02 | 1.27 |
| PD-1 | CD4^+^ naive T-cells | 0.58 | 6.0E-03 | 0.02 | 1.0E-02 | 7.0E-03 | 1.06 |
| PD-1 | CD4^+^ T-cells | 0.48 | 2.7E-02 | 0.03 | 3.4E-02 | 2.8E-02 | 1.19 |
| PD-1 | PD-1-high proportion | 0.37 | 9.4E-02 | 0.1 | 4.6E-01 | 3.7E-01 | 4.21 |
| PD-1 | IFN-gamma signature | 0.33 | 1.5E-01 | 0.16 | 4.0E-01 | 3.1E-01 | 3.38 |
| PD-1 | Cytolytic index | 0.25 | 2.8E-01 | 0.28 | 5.5E-01 | 4.0E-01 | 5.29 |
| PD-1-high proportion | CD4^+^ naive T-cells | 0.45 | 3.8E-02 | 0.046 | 4.5E-02 | 3.4E-02 | 1.2 |
| PD-1-high proportion | IFN-gamma signature | 0.37 | 9.8E-02 | 0.11 | 1.3E-01 | 1.2E-01 | 1.62 |
| PD-1-high proportion | CD4^+^ T-cells | 0.37 | 1.0E-01 | 0.11 | 5.3E-02 | 5.1E-02 | 1.28 |
| PD-1-high proportion | Cytolytic index | 0.32 | 1.6E-01 | 0.16 | 2.0E-01 | 1.9E-01 | 2 |
| CD4^+^ T-cells | IFN-gamma signature | 0.56 | 8.9E-03 | 0.02 | 7.6E-03 | 7.4E-03 | 1.05 |
| CD4^+^ T-cells | CD4^+^ naive T-cells | 0.51 | 1.9E-02 | 0.03 | 5.7E-02 | 4.5E-02 | 1.25 |
| CD4^+^ T-cells | Cytolytic index | 0.5 | 2.0E-02 | 0.03 | 1.9E-02 | 1.8E-02 | 1.11 |
| IFN-gamma signature | CD4^+^ naive T-cells | 0.58 | 5.6E-03 | 0.02 | 4.0E-03 | 3.3E-03 | 1.02 |
| IFN-gamma signature | Cytolytic index | 0.4 | 7.5E-02 | 0.09 | 5.5E-01 | 5.5E-01 | 7.12 |
| CD4^+^ naive T-cells | Cytolytic index | 0.53 | 1.4E-02 | 0.02 | 1.4E-02 | 1.8E-02 | 1.09 |

Abbreviation: *R*, Pearson correlation coefficient; LRT, log-likelihood ratio test; VIF, variance inflation factor.

**Supplementary Table S3. Evaluation of Trivariate Models for Seven Immune-Related Adverse Events-Associated Immunogenomic Factors^a^**

| **Bivariate model** | **Added variable** | ***R*** | ***P*** | **FDR** | **LRT *P* for trivariate vs bivariate models** | **VIF for variable 1** | **VIF for variable 2** | **VIF for variable 3** |
| --- | --- | --- | --- | --- | --- | --- | --- | --- |
| DC + TMB | CD4^+^ naive T-cells | 0.81 | 1.1E-05 | 1.1E-04 | 8.7E-04 | 1.20 | 1.08 | 1.11 |
| DC + TMB | CD4^+^ T-cells | 0.74 | 1.3E-04 | 4.4E-04 | 1.4E-02 | 1.08 | 1.29 | 1.24 |
| DC + TMB | CD8^+^ T-cells | 0.74 | 1.3E-04 | 4.4E-04 | 6.7E-02 | 1.41 | 1.09 | 1.37 |
| DC + TMB | Mast cells | 0.70 | 3.9E-04 | 8.5E-04 | 2.3E-01 | 1.40 | 1.13 | 1.45 |
| DC + TMB | Expanded immune signature | 0.69 | 5.6E-04 | 8.5E-04 | 9.0E-01 | 1.96 | 1.27 | 2.30 |
| DC + TMB | Cytolytic index | 0.69 | 5.9E-04 | 8.5E-04 | 7.6E-01 | 1.67 | 1.16 | 1.79 |
| DC + TMB | IFN-gamma signature | 0.69 | 6.0E-04 | 8.5E-04 | 8.2E-01 | 1.58 | 1.33 | 1.95 |
| DC + TMB | PD-L1 | 0.68 | 7.1E-04 | 8.9E-04 | 9.9E-01 | 1.59 | 1.32 | 1.95 |
| DC + TMB | PD-1 | 0.67 | 1.0E-03 | 1.1E-03 | 8.2E-01 | 1.70 | 1.18 | 1.86 |
| DC + TMB | PD-1-high proportion | 0.62 | 2.6E-03 | 2.6E-03 | 3.8E-01 | 1.46 | 1.14 | 1.53 |
| TMB + CD4^+^ naive T-cells | DC | 0.81 | 1.1E-05 | 1.1E-04 | 2.8E-04 | 1.08 | 1.11 | 1.20 |
| TMB + CD4^+^ naive T-cells | Mast cells | 0.76 | 5.5E-05 | 2.2E-04 | 7.5E-02 | 1.12 | 1.15 | 1.28 |
| TMB + CD4^+^ naive T-cells | Expanded immune signature | 0.75 | 8.4E-05 | 2.2E-04 | 2.4E-02 | 1.26 | 1.05 | 1.32 |
| TMB + CD4^+^ naive T-cells | PD-1 | 0.75 | 8.8E-05 | 2.2E-04 | 4.8E-02 | 1.18 | 1.06 | 1.24 |
| TMB + CD4^+^ naive T-cells | IFN-gamma signature | 0.73 | 1.5E-04 | 2.6E-04 | 7.3E-02 | 1.33 | 1.02 | 1.35 |
| TMB + CD4^+^ naive T-cells | Cytolytic index | 0.73 | 1.6E-04 | 2.6E-04 | 8.4E-02 | 1.16 | 1.09 | 1.26 |
| TMB + CD4^+^ naive T-cells | PD-L1 | 0.72 | 2.2E-04 | 3.0E-04 | 1.4E-01 | 1.33 | 1.07 | 1.41 |
| TMB + CD4^+^ naive T-cells | CD8^+^ T-cells | 0.72 | 2.4E-04 | 3.0E-04 | 2.3E-01 | 1.06 | 1.79 | 1.87 |
| TMB + CD4^+^ naive T-cells | CD4^+^ T-cells | 0.71 | 3.3E-04 | 3.7E-04 | 5.0E-01 | 1.27 | 1.29 | 1.59 |
| TMB + CD4^+^ naive T-cells | PD-1-high proportion | 0.69 | 5.1E-04 | 5.1E-04 | 1.7E-01 | 1.14 | 1.21 | 1.37 |

Abbreviation: *R*, Pearson correlation coefficient; LRT, log-likelihood ratio test; VIF, variance inflation factor.

^a^Variables 1 and 2 indicate DC and TMB in the DC-TMB-based trivariate model, respectively, but TMB and CD4^+^ naive T-cells in the TMB-CD4^+^ naive T-cells-based trivariate model. Variable 3 indicates the added variable.

**Supplementary Table S4. Evaluation of Bivariate Models for 11 Immune-Related Adverse Events-Associated Novel Molecular Factors**

| **Variable 1** | **Variable 2** | ***R*** | ***P*** | **FDR** | **LRT *P* for variable 1** | **LRT *P* for variable 2** | **VIF** |
| --- | --- | --- | --- | --- | --- | --- | --- |
| *IRF4* | *TCL1A* | 0.854 | 8.5E-07 | 2.3E-05 | 1.6E-02 | 3.2E-03 | 2.52 |
| *IRF4* | *GPNMB* | 0.84 | 1.6E-06 | 2.3E-05 | 3.1E-02 | 3.3E-03 | 2.61 |
| *IRF4* | *FAIM3* | 0.82 | 5.4E-06 | 2.3E-05 | 6.6E-02 | 4.9E-03 | 2.90 |
| *IRF4* | SHC-pY317 | 0.84 | 2.3E-06 | 2.3E-05 | 2.3E-02 | 1.3E-04 | 1.78 |
| *IRF4* | let-7i-5p | 0.80 | 1.3E-05 | 3.1E-05 | 2.8E-01 | 5.9E-03 | 3.61 |
| *IRF4* | miR-4709-3p | 0.82 | 6.8E-06 | 2.5E-05 | 1.1E-01 | 1.7E-03 | 2.60 |
| *IRF4* | miR-629-5p | 0.80 | 1.6E-05 | 3.7E-05 | 5.2E-01 | 3.2E-03 | 3.70 |
| *IRF4* | miR-511-5p | 0.82 | 6.1E-06 | 2.4E-05 | 1.9E-01 | 8.6E-04 | 2.47 |
| *IRF4* | miR-155-3p | 0.84 | 2.5E-06 | 2.3E-05 | 7.1E-02 | 2.0E-04 | 1.91 |
| *IRF4* | miR-3136-5p | 0.80 | 1.1E-05 | 2.8E-05 | 4.3E-01 | 4.7E-04 | 2.50 |
| *TCL1A* | *GPNMB* | 0.83 | 3.1E-06 | 2.3E-05 | 3.7E-03 | 2.0E-03 | 1.99 |
| *TCL1A* | *FAIM3* | 0.77 | 4.3E-05 | 6.0E-05 | 4.9E-02 | 1.8E-02 | 2.98 |
| *TCL1A* | SHC-pY317 | 0.83 | 3.0E-06 | 2.3E-05 | 4.0E-03 | 1.1E-04 | 1.53 |
| *TCL1A* | let-7i-5p | 0.83 | 3.7E-06 | 2.3E-05 | 5.1E-03 | 7.4E-04 | 1.81 |
| *TCL1A* | miR-4709-3p | 0.79 | 2.3E-05 | 4.1E-05 | 4.3E-02 | 3.4E-03 | 2.28 |
| *TCL1A* | miR-629-5p | 0.81 | 8.9E-06 | 2.8E-05 | 2.1E-02 | 1.0E-03 | 1.91 |
| *TCL1A* | miR-511-5p | 0.76 | 6.7E-05 | 8.6E-05 | 4.0E-01 | 7.0E-03 | 3.40 |
| *TCL1A* | miR-155-3p | 0.79 | 2.4E-05 | 4.1E-05 | 1.4E-01 | 1.6E-03 | 2.21 |
| *TCL1A* | miR-3136-5p | 0.78 | 3.3E-05 | 5.1E-05 | 1.2E-01 | 8.2E-04 | 2.00 |
| *GPNMB* | *FAIM3* | 0.77 | 4.0E-05 | 5.8E-05 | 2.8E-02 | 2.0E-02 | 2.72 |
| *GPNMB* | SHC-pY317 | 0.81 | 9.9E-06 | 2.8E-05 | 1.7E-02 | 8.1E-04 | 1.75 |
| *GPNMB* | let-7i-5p | 0.77 | 3.8E-05 | 5.7E-05 | 9.4E-02 | 2.2E-02 | 3.11 |
| *GPNMB* | miR-4709-3p | 0.83 | 2.8E-06 | 2.3E-05 | 4.1E-03 | 6.6E-04 | 1.69 |
| *GPNMB* | miR-629-5p | 0.82 | 5.2E-06 | 2.3E-05 | 2.4E-02 | 2.1E-03 | 1.96 |
| *GPNMB* | miR-511-5p | 0.76 | 6.5E-05 | 8.5E-05 | 9.1E-02 | 3.9E-03 | 2.29 |
| *GPNMB* | miR-155-3p | 0.79 | 2.4E-05 | 4.1E-05 | 4.8E-02 | 1.2E-03 | 1.87 |
| *GPNMB* | miR-3136-5p | 0.81 | 1.0E-05 | 2.8E-05 | 1.9E-02 | 2.9E-04 | 1.58 |
| *FAIM3* | SHC-pY317 | 0.82 | 4.3E-06 | 2.3E-05 | 1.1E-03 | 7.9E-05 | 1.40 |
| *FAIM3* | let-7i-5p | 0.79 | 2.5E-05 | 4.1E-05 | 2.0E-02 | 7.0E-03 | 2.20 |
| *FAIM3* | miR-4709-3p | 0.78 | 2.6E-05 | 4.3E-05 | 2.8E-02 | 5.8E-03 | 2.18 |
| *FAIM3* | miR-629-5p | 0.81 | 1.0E-05 | 2.8E-05 | 7.2E-03 | 9.3E-04 | 1.70 |
| *FAIM3* | miR-511-5p | 0.75 | 8.7E-05 | 1.0E-04 | 3.3E-02 | 2.1E-03 | 1.92 |
| *FAIM3* | miR-155-3p | 0.75 | 8.1E-05 | 1.0E-04 | 6.3E-02 | 2.1E-03 | 1.97 |
| *FAIM3* | miR-3136-5p | 0.79 | 2.2E-05 | 4.1E-05 | 1.4E-02 | 2.9E-04 | 1.53 |
| SHC-pY317 | let-7i-5p | 0.77 | 4.8E-05 | 6.5E-05 | 2.7E-03 | 1.5E-02 | 1.75 |
| SHC-pY317 | miR-4709-3p | 0.80 | 1.3E-05 | 3.1E-05 | 9.5E-04 | 3.1E-03 | 1.49 |
| SHC-pY317 | miR-629-5p | 0.83 | 3.7E-06 | 2.3E-05 | 3.3E-04 | 6.1E-04 | 1.33 |
| SHC-pY317 | miR-511-5p | 0.81 | 8.6E-06 | 2.8E-05 | 4.6E-04 | 4.7E-04 | 1.30 |
| SHC-pY317 | miR-155-3p | 0.78 | 2.8E-05 | 4.4E-05 | 1.3E-03 | 7.3E-04 | 1.33 |
| SHC-pY317 | miR-3136-5p | 0.73 | 1.8E-04 | 1.9E-04 | 1.6E-02 | 4.8E-03 | 1.59 |
| let-7i-5p | miR-4709-3p | 0.79 | 2.3E-05 | 4.1E-05 | 1.2E-02 | 7.1E-03 | 1.95 |
| let-7i-5p | miR-629-5p | 0.79 | 2.4E-05 | 4.1E-05 | 2.4E-02 | 8.1E-03 | 2.04 |
| let-7i-5p | miR-511-5p | 0.77 | 4.3E-05 | 6.0E-05 | 1.5E-02 | 2.9E-03 | 1.76 |
| let-7i-5p | miR-155-3p | 0.74 | 1.1E-04 | 1.3E-04 | 4.0E-02 | 3.7E-03 | 1.88 |
| let-7i-5p | miR-3136-5p | 0.75 | 9.8E-05 | 1.1E-04 | 6.4E-02 | 3.3E-03 | 1.89 |
| miR-4709-3p | miR-629-5p | 0.82 | 4.8E-06 | 2.3E-05 | 2.1E-03 | 1.2E-03 | 1.52 |
| miR-4709-3p | miR-511-5p | 0.79 | 2.2E-05 | 4.1E-05 | 1.5E-02 | 4.7E-03 | 1.78 |
| miR-4709-3p | miR-155-3p | 0.79 | 2.0E-05 | 4.1E-05 | 7.2E-03 | 1.2E-03 | 1.52 |
| miR-4709-3p | miR-3136-5p | 0.75 | 8.4E-05 | 1.0E-04 | 7.3E-02 | 6.1E-03 | 1.97 |
| miR-629-5p | miR-511-5p | 0.74 | 1.5E-04 | 1.6E-04 | 2.9E-02 | 1.6E-02 | 2.03 |
| miR-629-5p | miR-155-3p | 0.71 | 3.1E-04 | 3.3E-04 | 7.6E-02 | 2.0E-02 | 2.26 |
| miR-629-5p | miR-3136-5p | 0.68 | 6.2E-04 | 6.3E-04 | 2.4E-01 | 3.0E-02 | 2.90 |
| miR-511-5p | miR-155-3p | 0.74 | 1.2E-04 | 1.3E-04 | 1.3E-02 | 6.9E-03 | 1.65 |
| miR-511-5p | miR-3136-5p | 0.71 | 3.6E-04 | 3.7E-04 | 5.2E-02 | 1.4E-02 | 1.94 |
| miR-155-3p | miR-3136-5p | 0.64 | 1.8E-03 | 1.8E-03 | 1.2E-01 | 6.3E-02 | 2.62 |

Abbreviation: *R*, Pearson correlation coefficient; LRT, log-likelihood ratio test; VIF, variance inflation factor.

**Supplementary Table S5. Evaluation of Trivariate Models for *IRF4* and *TCL1A* Together With Nine Other Immune-Related Adverse Events-Associated Molecular Factors^a^**

| **Bivariate model** | **Added variable** | ***R*** | ***P*** | **FDR** | **LRT *P* for trivariate vs bivariate models** | **VIF for variable 1** | **VIF for variable 2** | **VIF for variable 3** |
| --- | --- | --- | --- | --- | --- | --- | --- | --- |
| *IRF4* + *TCL1A* | *GPNMB* | 0.86 | 6.23E-07 | 2.54E-06 | 0.07 | 3.51 | 2.68 | 2.77 |
| *IRF4* + *TCL1A* | *FAIM3* | 0.82 | 4.40E-06 | 4.40E-06 | 0.40 | 3.29 | 3.38 | 3.89 |
| *IRF4* + *TCL1A* | SHC-pY317 | 0.87 | 3.06E-07 | 2.54E-06 | 0.03 | 3.00 | 2.59 | 1.82 |
| *IRF4* + *TCL1A* | let-7i-5p | 0.84 | 1.58E-06 | 2.92E-06 | 0.23 | 5.04 | 2.52 | 3.61 |
| *IRF4* + *TCL1A* | miR-4709-3p | 0.85 | 8.48E-07 | 2.54E-06 | 0.37 | 3.30 | 2.89 | 2.98 |
| *IRF4* + *TCL1A* | miR-629-5p | 0.84 | 2.42E-06 | 3.18E-06 | 0.60 | 4.92 | 2.54 | 3.73 |
| *IRF4* + *TCL1A* | miR-511-5p | 0.84 | 2.47E-06 | 3.18E-06 | 0.91 | 2.87 | 3.95 | 3.86 |
| *IRF4* + *TCL1A* | miR-155-3p | 0.83 | 3.13E-06 | 3.52E-06 | 0.35 | 2.72 | 3.15 | 2.39 |
| *IRF4* + *TCL1A* | miR-3136-5p | 0.84 | 1.62E-06 | 2.92E-06 | 0.82 | 3.39 | 2.71 | 2.68 |

Abbreviation: *R*, Pearson correlation coefficient; LRT, log-likelihood ratio test; VIF, variance inflation factor.

^a^Variables 1, 2, and 3 indicate *IRF4*, *TCL1A*, and the added variable in the *IRF4*-*TCL1A*-based trivariate model, respectively.

**Supplementary Table S6. Evaluation of Combination of Immune-Related Adverse Events-Associated Molecular and Immunogenomic Factors**

| **Variable 1** | **Variable 2** | ***R*** | ***P*** | **FDR** | **LRT *P* for variable 1** | **LRT *P* for variable 2** | **VIF** |
| --- | --- | --- | --- | --- | --- | --- | --- |
| *IRF4* | DC | 0.83 | 3.6E-06 | 1.1E-04 | 0.005 | 5.4E-06 | 1.37 |
| *IRF4* | TMB | 0.80 | 1.6E-05 | 1.2E-04 | 0.74 | 5.5E-05 | 1.99 |
| *IRF4* | Expanded immune signature | 0.81 | 7.8E-06 | 1.1E-04 | 0.44 | 3.7E-05 | 1.74 |
| *IRF4* | PD-L1 | 0.83 | 4.0E-06 | 1.1E-04 | 0.17 | 8.1E-06 | 1.38 |
| *IRF4* | CD8^+^ T-cells | 0.84 | 2.0E-06 | 1.1E-04 | 0.05 | 2.9E-06 | 1.26 |
| *IRF4* | Mast cells | 0.80 | 1.6E-05 | 1.2E-04 | 0.91 | 1.8E-05 | 1.87 |
| *IRF4* | PD-1 | 0.79 | 2.2E-05 | 1.4E-04 | 0.85 | 1.7E-05 | 1.73 |
| *IRF4* | PD-1-high proportion | 0.76 | 6.5E-05 | 2.9E-04 | 0.61 | 1.3E-05 | 1.58 |
| *IRF4* | CD4^+^ T-cells | 0.79 | 2.3E-05 | 1.4E-04 | 0.99 | 1.5E-05 | 1.76 |
| *IRF4* | IFN-gamma signature | 0.81 | 9.9E-06 | 1.1E-04 | 0.59 | 1.2E-05 | 1.55 |
| *IRF4* | CD4^+^ naive T-cells | 0.83 | 4.1E-06 | 1.1E-04 | 0.06 | 1.9E-06 | 1.21 |
| *IRF4* | Cytolytic index | 0.81 | 7.1E-06 | 1.1E-04 | 0.33 | 8.9E-06 | 1.43 |
| *TCL1A* | DC | 0.70 | 4.5E-04 | 7.0E-04 | 0.73 | 1.4E-03 | 2.90 |
| *TCL1A* | TMB | 0.80 | 1.2E-05 | 1.1E-04 | 0.04 | 2.9E-05 | 1.35 |
| *TCL1A* | Expanded immune signature | 0.76 | 5.7E-05 | 2.8E-04 | 0.79 | 2.2E-04 | 2.53 |
| *TCL1A* | PD-L1 | 0.77 | 4.6E-05 | 2.4E-04 | 0.41 | 6.8E-05 | 1.57 |
| *TCL1A* | CD8^+^ T-cells | 0.81 | 6.9E-06 | 1.1E-04 | 0.03 | 7.6E-06 | 1.21 |
| *TCL1A* | Mast cells | 0.78 | 3.2E-05 | 1.8E-04 | 0.12 | 2.3E-05 | 1.32 |
| *TCL1A* | PD-1 | 0.75 | 9.1E-05 | 3.4E-04 | 0.73 | 7.3E-05 | 2.10 |
| *TCL1A* | PD-1-high proportion | 0.71 | 3.6E-04 | 6.1E-04 | 0.71 | 6.4E-05 | 1.68 |
| *TCL1A* | CD4^+^ T-cells | 0.76 | 7.1E-05 | 3.0E-04 | 0.11 | 1.8E-05 | 1.29 |
| *TCL1A* | IFN-gamma signature | 0.76 | 7.5E-05 | 3.1E-04 | 0.96 | 6.5E-05 | 1.87 |
| *TCL1A* | CD4^+^ naive T-cells | 0.82 | 4.8E-06 | 1.1E-04 | 0.009 | 1.5E-06 | 1.11 |
| *TCL1A* | Cytolytic index | 0.76 | 7.1E-05 | 3.0E-04 | 0.98 | 6.5E-05 | 1.86 |
| *GPNMB* | DC | 0.75 | 9.5E-05 | 3.4E-04 | 0.01 | 8.7E-05 | 1.41 |
| *GPNMB* | TMB | 0.80 | 1.3E-05 | 1.2E-04 | 0.06 | 6.8E-05 | 1.38 |
| *GPNMB* | Expanded immune signature | 0.71 | 3.0E-04 | 5.3E-04 | 0.28 | 2.2E-04 | 1.67 |
| *GPNMB* | PD-L1 | 0.73 | 1.7E-04 | 4.3E-04 | 0.62 | 1.5E-04 | 1.74 |
| *GPNMB* | CD8^+^ T-cells | 0.81 | 9.7E-06 | 1.1E-04 | 0.03 | 1.3E-05 | 1.20 |
| *GPNMB* | Mast cells | 0.75 | 8.4E-05 | 3.4E-04 | 0.61 | 1.3E-04 | 1.69 |
| *GPNMB* | PD-1 | 0.72 | 2.3E-04 | 4.7E-04 | 0.34 | 8.7E-05 | 1.50 |
| *GPNMB* | PD-1-high proportion | 0.71 | 2.7E-04 | 4.9E-04 | 0.23 | 5.7E-05 | 1.39 |
| *GPNMB* | CD4^+^ T-cells | 0.79 | 2.2E-05 | 1.4E-04 | 0.10 | 2.9E-05 | 1.28 |
| *GPNMB* | IFN-gamma signature | 0.72 | 2.5E-04 | 4.8E-04 | 0.69 | 1.1E-04 | 1.68 |
| *GPNMB* | CD4^+^ naive T-cells | 0.79 | 2.3E-05 | 1.4E-04 | 0.10 | 2.4E-05 | 1.25 |
| *GPNMB* | Cytolytic index | 0.72 | 2.6E-04 | 4.8E-04 | 0.46 | 8.8E-05 | 1.54 |
| *FAIM3* | DC | 0.70 | 4.6E-04 | 7.1E-04 | 0.06 | 5.4E-04 | 1.66 |
| *FAIM3* | TMB | 0.81 | 8.1E-06 | 1.1E-04 | 0.004 | 8.5E-06 | 1.18 |
| *FAIM3* | Expanded immune signature | 0.67 | 9.6E-04 | 1.2E-03 | 0.93 | 5.7E-04 | 2.36 |
| *FAIM3* | PD-L1 | 0.72 | 2.1E-04 | 4.6E-04 | 0.17 | 8.8E-05 | 1.40 |
| *FAIM3* | CD8^+^ T-cells | 0.75 | 1.0E-04 | 3.5E-04 | 0.07 | 4.1E-05 | 1.28 |
| *FAIM3* | Mast cells | 0.72 | 2.1E-04 | 4.6E-04 | 0.69 | 1.8E-04 | 2.27 |
| *FAIM3* | PD-1 | 0.66 | 1.1E-03 | 1.4E-03 | 0.60 | 1.7E-04 | 1.69 |
| *FAIM3* | PD-1-high proportion | 0.68 | 6.9E-04 | 9.8E-04 | 0.20 | 7.2E-05 | 1.38 |
| *FAIM3* | CD4^+^ T-cells | 0.73 | 1.5E-04 | 4.1E-04 | 0.12 | 4.7E-05 | 1.30 |
| *FAIM3* | IFN-gamma signature | 0.68 | 6.9E-04 | 9.8E-04 | 0.95 | 1.6E-04 | 1.94 |
| *FAIM3* | CD4^+^ naive T-cells | 0.72 | 2.2E-04 | 4.6E-04 | 0.44 | 9.6E-05 | 1.50 |
| *FAIM3* | Cytolytic index | 0.66 | 1.2E-03 | 1.5E-03 | 0.91 | 1.6E-04 | 1.85 |
| SHC-pY317 | DC | 0.70 | 4.1E-04 | 6.5E-04 | 0.009 | 1.4E-03 | 1.39 |
| SHC-pY317 | TMB | 0.72 | 2.6E-04 | 4.8E-04 | 0.05 | 1.3E-03 | 1.40 |
| SHC-pY317 | Expanded immune signature | 0.73 | 1.7E-04 | 4.3E-04 | 0.006 | 1.4E-04 | 1.16 |
| SHC-pY317 | PD-L1 | 0.71 | 3.4E-04 | 6.0E-04 | 0.03 | 3.0E-04 | 1.21 |
| SHC-pY317 | CD8^+^ T-cells | 0.80 | 1.1E-05 | 1.1E-04 | 0.0003 | 3.2E-06 | 1.03 |
| SHC-pY317 | Mast cells | 0.68 | 7.3E-04 | 1.0E-03 | 0.09 | 5.7E-04 | 1.30 |
| SHC-pY317 | PD-1 | 0.69 | 4.9E-04 | 7.5E-04 | 0.03 | 2.3E-04 | 1.19 |
| SHC-pY317 | PD-1-high proportion | 0.68 | 7.4E-04 | 1.0E-03 | 0.02 | 1.1E-04 | 1.13 |
| SHC-pY317 | CD4^+^ T-cells | 0.71 | 3.1E-04 | 5.4E-04 | 0.03 | 1.7E-04 | 1.16 |
| SHC-pY317 | IFN-gamma signature | 0.72 | 2.1E-04 | 4.6E-04 | 0.009 | 5.8E-05 | 1.10 |
| SHC-pY317 | CD4^+^ naive T-cells | 0.74 | 1.4E-04 | 4.1E-04 | 0.007 | 3.7E-05 | 1.08 |
| SHC-pY317 | Cytolytic index | 0.72 | 2.6E-04 | 4.8E-04 | 0.009 | 6.1E-05 | 1.10 |
| let-7i-5p | DC | 0.75 | 9.0E-05 | 3.4E-04 | 0.002 | 6.2E-05 | 1.26 |
| let-7i-5p | TMB | 0.74 | 1.2E-04 | 3.6E-04 | 0.10 | 4.0E-04 | 1.48 |
| let-7i-5p | Expanded immune signature | 0.74 | 1.1E-04 | 3.6E-04 | 0.05 | 1.9E-04 | 1.35 |
| let-7i-5p | PD-L1 | 0.68 | 7.0E-04 | 9.9E-04 | 0.48 | 4.9E-04 | 1.71 |
| let-7i-5p | CD8^+^ T-cells | 0.74 | 1.5E-04 | 4.1E-04 | 0.19 | 2.5E-04 | 1.43 |
| let-7i-5p | Mast cells | 0.73 | 1.8E-04 | 4.3E-04 | 0.16 | 1.9E-04 | 1.38 |
| let-7i-5p | PD-1 | 0.73 | 2.0E-04 | 4.6E-04 | 0.13 | 1.5E-04 | 1.34 |
| let-7i-5p | PD-1-high proportion | 0.68 | 7.4E-04 | 1.0E-03 | 0.18 | 1.7E-04 | 1.37 |
| let-7i-5p | CD4^+^ T-cells | 0.70 | 4.1E-04 | 6.5E-04 | 0.67 | 4.0E-04 | 1.75 |
| let-7i-5p | IFN-gamma signature | 0.74 | 1.2E-04 | 3.6E-04 | 0.07 | 7.4E-05 | 1.24 |
| let-7i-5p | CD4^+^ naive T-cells | 0.72 | 2.1E-04 | 4.6E-04 | 0.42 | 2.5E-04 | 1.51 |
| let-7i-5p | Cytolytic index | 0.74 | 1.2E-04 | 3.6E-04 | 0.09 | 9.1E-05 | 1.26 |
| miR-4709-3p | DC | 0.49 | 2.5E-02 | 2.5E-02 | 0.03 | 1.2E-03 | 1.53 |
| miR-4709-3p | TMB | 0.77 | 4.5E-05 | 2.4E-04 | 0.04 | 3.4E-04 | 1.36 |
| miR-4709-3p | Expanded immune signature | 0.73 | 1.5E-04 | 4.1E-04 | 0.07 | 4.2E-04 | 1.40 |
| miR-4709-3p | PD-L1 | 0.79 | 1.8E-05 | 1.3E-04 | 0.008 | 2.7E-05 | 1.13 |
| miR-4709-3p | CD8^+^ T-cells | 0.77 | 4.7E-05 | 2.4E-04 | 0.04 | 1.1E-04 | 1.23 |
| miR-4709-3p | Mast cells | 0.75 | 9.2E-05 | 3.4E-04 | 0.15 | 3.0E-04 | 1.38 |
| miR-4709-3p | PD-1 | 0.72 | 2.6E-04 | 4.8E-04 | 0.53 | 6.9E-04 | 1.72 |
| miR-4709-3p | PD-1-high proportion | 0.66 | 1.2E-03 | 1.5E-03 | 0.83 | 7.3E-04 | 2.27 |
| miR-4709-3p | CD4^+^ T-cells | 0.72 | 2.4E-04 | 4.7E-04 | 0.29 | 4.0E-04 | 1.48 |
| miR-4709-3p | IFN-gamma signature | 0.74 | 1.4E-04 | 4.1E-04 | 0.10 | 1.6E-04 | 1.28 |
| miR-4709-3p | CD4^+^ naive T-cells | 0.73 | 1.9E-04 | 4.6E-04 | 0.26 | 2.9E-04 | 1.41 |
| miR-4709-3p | Cytolytic index | 0.74 | 1.5E-04 | 4.1E-04 | 0.12 | 2.0E-04 | 1.31 |
| miR-629-5p | DC | 0.73 | 1.8E-04 | 4.3E-04 | 0.004 | 3.0E-04 | 1.29 |
| miR-629-5p | TMB | 0.70 | 3.9E-04 | 6.5E-04 | 0.20 | 2.1E-03 | 1.71 |
| miR-629-5p | Expanded immune signature | 0.69 | 5.1E-04 | 7.7E-04 | 0.11 | 1.1E-03 | 1.50 |
| miR-629-5p | PD-L1 | 0.71 | 3.5E-04 | 6.0E-04 | 0.08 | 3.4E-04 | 1.30 |
| miR-629-5p | CD8^+^ T-cells | 0.73 | 1.6E-04 | 4.2E-04 | 0.05 | 2.1E-04 | 1.24 |
| miR-629-5p | Mast cells | 0.68 | 7.5E-04 | 1.0E-03 | 0.38 | 9.8E-04 | 1.62 |
| miR-629-5p | PD-1 | 0.67 | 8.1E-04 | 1.1E-03 | 0.26 | 7.2E-04 | 1.49 |
| miR-629-5p | PD-1-high proportion | 0.64 | 1.8E-03 | 2.0E-03 | 0.19 | 4.9E-04 | 1.39 |
| miR-629-5p | CD4^+^ T-cells | 0.72 | 2.1E-04 | 4.6E-04 | 0.08 | 2.4E-04 | 1.26 |
| miR-629-5p | IFN-gamma signature | 0.69 | 6.1E-04 | 9.0E-04 | 0.25 | 5.8E-04 | 1.45 |
| miR-629-5p | CD4^+^ naive T-cells | 0.82 | 4.7E-06 | 1.1E-04 | 0.001 | 3.1E-06 | 1.03 |
| miR-629-5p | Cytolytic index | 0.70 | 4.1E-04 | 6.5E-04 | 0.11 | 3.1E-04 | 1.30 |
| miR-511-5p | DC | 0.62 | 2.8E-03 | 3.0E-03 | 0.10 | 1.2E-02 | 1.97 |
| miR-511-5p | TMB | 0.68 | 6.9E-04 | 9.8E-04 | 0.21 | 3.9E-03 | 1.76 |
| miR-511-5p | Expanded immune signature | 0.62 | 2.8E-03 | 3.0E-03 | 0.47 | 6.1E-03 | 2.17 |
| miR-511-5p | PD-L1 | 0.66 | 1.3E-03 | 1.5E-03 | 0.17 | 1.2E-03 | 1.46 |
| miR-511-5p | CD8^+^ T-cells | 0.72 | 2.3E-04 | 4.7E-04 | 0.03 | 2.8E-04 | 1.21 |
| miR-511-5p | Mast cells | 0.67 | 9.7E-04 | 1.2E-03 | 0.07 | 4.7E-04 | 1.27 |
| miR-511-5p | PD-1 | 0.62 | 2.8E-03 | 3.0E-03 | 0.59 | 2.3E-03 | 1.87 |
| miR-511-5p | PD-1-high proportion | 0.61 | 3.6E-03 | 3.8E-03 | 0.36 | 1.5E-03 | 1.59 |
| miR-511-5p | CD4^+^ T-cells | 0.68 | 7.6E-04 | 1.0E-03 | 0.10 | 5.1E-04 | 1.29 |
| miR-511-5p | IFN-gamma signature | 0.62 | 2.6E-03 | 2.9E-03 | 0.65 | 1.9E-03 | 1.86 |
| miR-511-5p | CD4^+^ naive T-cells | 0.74 | 1.2E-04 | 3.6E-04 | 0.01 | 6.9E-05 | 1.10 |
| miR-511-5p | Cytolytic index | 0.63 | 2.2E-03 | 2.5E-03 | 0.70 | 2.0E-03 | 1.91 |
| miR-155-3p | DC | 0.70 | 4.0E-04 | 6.5E-04 | 0.04 | 9.1E-03 | 1.66 |
| miR-155-3p | TMB | 0.72 | 2.4E-04 | 4.7E-04 | 0.01 | 6.5E-04 | 1.23 |
| miR-155-3p | Expanded immune signature | 0.65 | 1.4E-03 | 1.7E-03 | 0.55 | 1.3E-02 | 2.47 |
| miR-155-3p | PD-L1 | 0.66 | 1.2E-03 | 1.5E-03 | 0.28 | 3.3E-03 | 1.63 |
| miR-155-3p | CD8^+^ T-cells | 0.67 | 9.5E-04 | 1.2E-03 | 0.16 | 2.1E-03 | 1.45 |
| miR-155-3p | Mast cells | 0.66 | 1.3E-03 | 1.5E-03 | 0.28 | 2.7E-03 | 1.57 |
| miR-155-3p | PD-1 | 0.61 | 3.6E-03 | 3.8E-03 | 0.88 | 5.0E-03 | 2.33 |
| miR-155-3p | PD-1-high proportion | 0.50 | 2.1E-02 | 2.1E-02 | 0.80 | 4.2E-03 | 2.14 |
| miR-155-3p | CD4^+^ T-cells | 0.63 | 2.3E-03 | 2.6E-03 | 0.07 | 6.9E-04 | 1.24 |
| miR-155-3p | IFN-gamma signature | 0.65 | 1.4E-03 | 1.6E-03 | 0.83 | 4.0E-03 | 2.16 |
| miR-155-3p | CD4^+^ naive T-cells | 0.67 | 8.9E-04 | 1.2E-03 | 0.04 | 3.8E-04 | 1.17 |
| miR-155-3p | Cytolytic index | 0.65 | 1.5E-03 | 1.8E-03 | 0.94 | 4.2E-03 | 2.47 |
| miR-3136-5p | DC | 0.68 | 7.0E-04 | 9.9E-04 | 0.005 | 2.4E-03 | 1.31 |
| miR-3136-5p | TMB | 0.62 | 2.5E-03 | 2.8E-03 | 0.20 | 1.3E-02 | 1.85 |
| miR-3136-5p | Expanded immune signature | 0.60 | 3.9E-03 | 4.1E-03 | 0.14 | 8.3E-03 | 1.65 |
| miR-3136-5p | PD-L1 | 0.69 | 5.3E-04 | 8.0E-04 | 0.01 | 3.3E-04 | 1.13 |
| miR-3136-5p | CD8^+^ T-cells | 0.72 | 2.2E-04 | 4.6E-04 | 0.009 | 2.5E-04 | 1.11 |
| miR-3136-5p | Mast cells | 0.64 | 1.8E-03 | 2.0E-03 | 0.10 | 2.2E-03 | 1.34 |
| miR-3136-5p | PD-1 | 0.57 | 7.0E-03 | 7.1E-03 | 0.54 | 7.3E-03 | 1.96 |
| miR-3136-5p | PD-1-high proportion | 0.50 | 2.2E-02 | 2.3E-02 | 0.59 | 6.6E-03 | 1.97 |
| miR-3136-5p | CD4^+^ T-cells | 0.60 | 3.7E-03 | 3.9E-03 | 0.25 | 3.6E-03 | 1.52 |
| miR-3136-5p | IFN-gamma signature | 0.60 | 4.2E-03 | 4.3E-03 | 0.21 | 3.1E-03 | 1.46 |
| miR-3136-5p | CD4^+^ naive T-cells | 0.76 | 6.1E-05 | 2.9E-04 | 0.002 | 3.4E-05 | 1.03 |
| miR-3136-5p | Cytolytic index | 0.60 | 4.2E-03 | 4.3E-03 | 0.24 | 3.4E-03 | 1.50 |

Abbreviation: *R*, Pearson correlation coefficient; LRT, log-likelihood ratio test; VIF, variance inflation factor.

**Supplementary Figure S1. Flow Chart Illustrating Main Analyses**

The 22 cancer types comprise breast, cervical, cholangio, colorectal, endometrial, esophageal, gastric, glioblastoma, glioma, head and neck, hepatocellular, lung adenocarcinoma, lung squamous cell, melanoma, mesothelioma, ovarian, pancreatic, prostate, renal, sarcoma, lung small cell, and urothelial cancers. The 21 common cancer types comprise all cancers above except lung small cell cancer. The US Food and Drug Administration’s Adverse Event Reporting System, FAERS; AE, adverse event; irAE, immune-related adverse event; ROR, reporting odds ratio; ORR, objective response rate; RPPA, reverse phase protein arrays; FDR, false discovery rate.

**Supplementary Figure S2. Reporting Odds Ratio of Immune-Related Adverse Event for Anti-PD-1 Therapy Across Cancer Types**


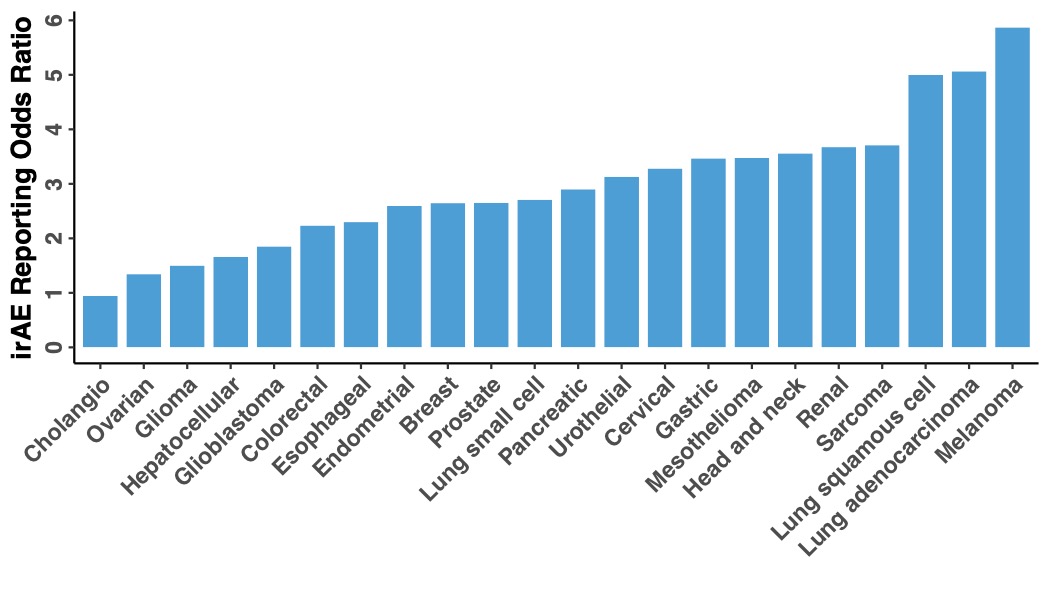
irAE, immune-related adverse event.

**Supplementary Figure S3. Correlation of Immune-Related Adverse Event Reporting Odds Ratio with Objective Response Rate During Anti-PD-1 Therapy Across Cancer Types**

**
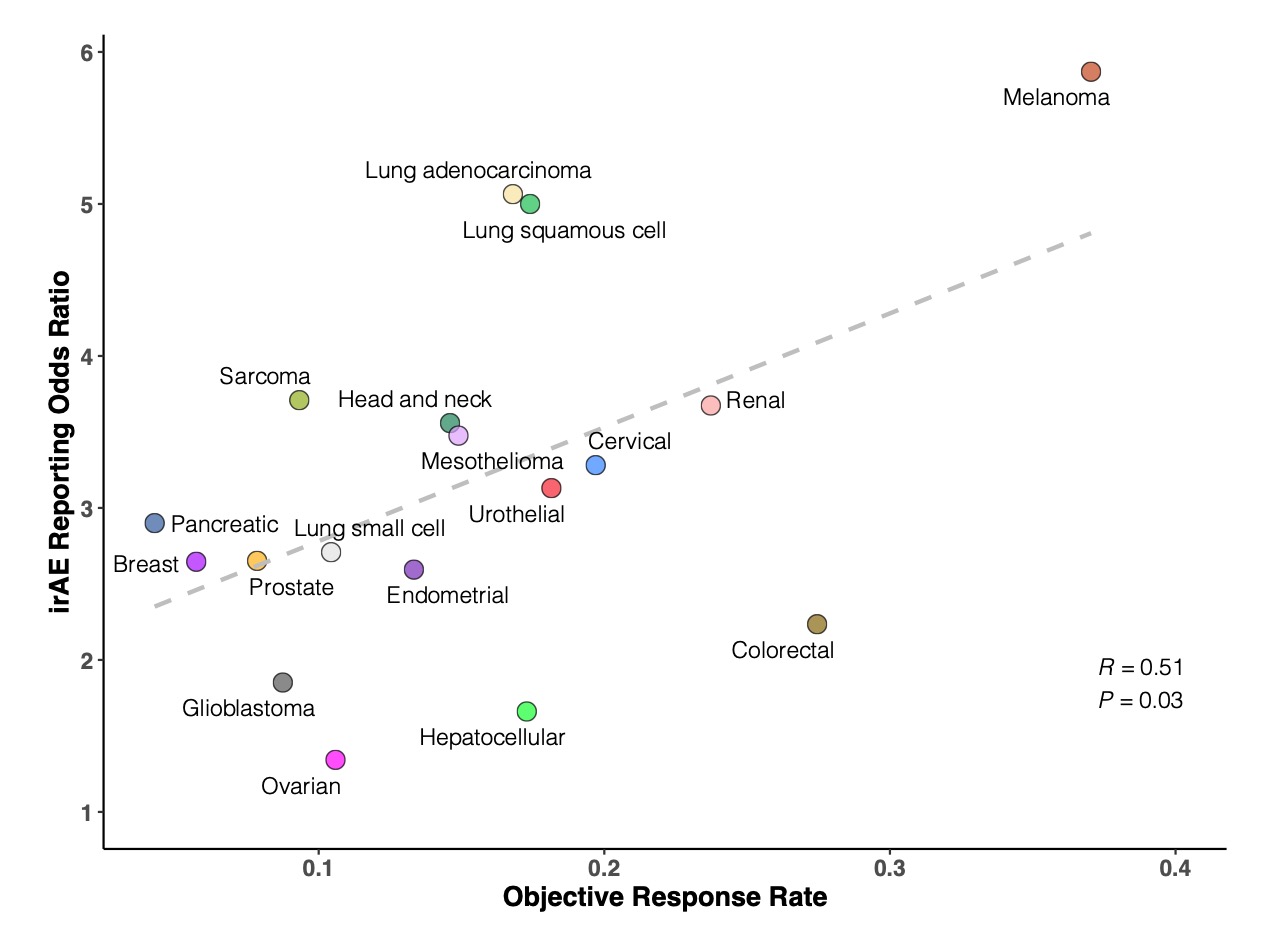
**

Cancer types are depicted in distinct colors. *R*, Pearson correlation coefficient; irAE, immune-related adverse event.

**Supplementary Figure S4. Immunogenomic Correlates of Immune-Related Adverse Event Reporting Odds Ratio During Anti-PD-1 Therapy Across Cancer Types**

**
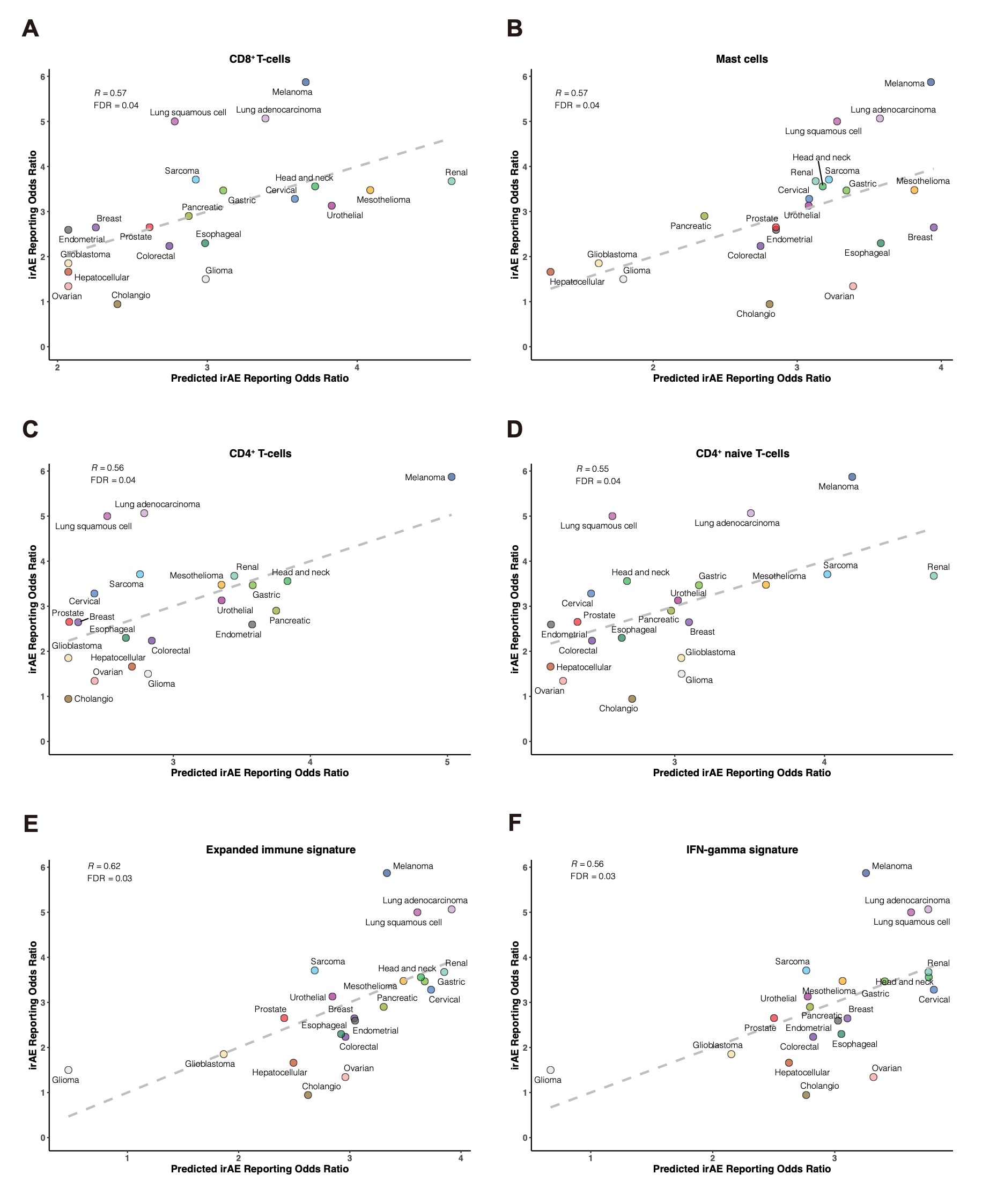
**

(*continued*)

**Supplementary Figure S4. Immunogenomic Correlates of Immune-Related Adverse Event Reporting Odds Ratio During Anti-PD-1 Therapy Across Cancer Types (*continued*)**

**
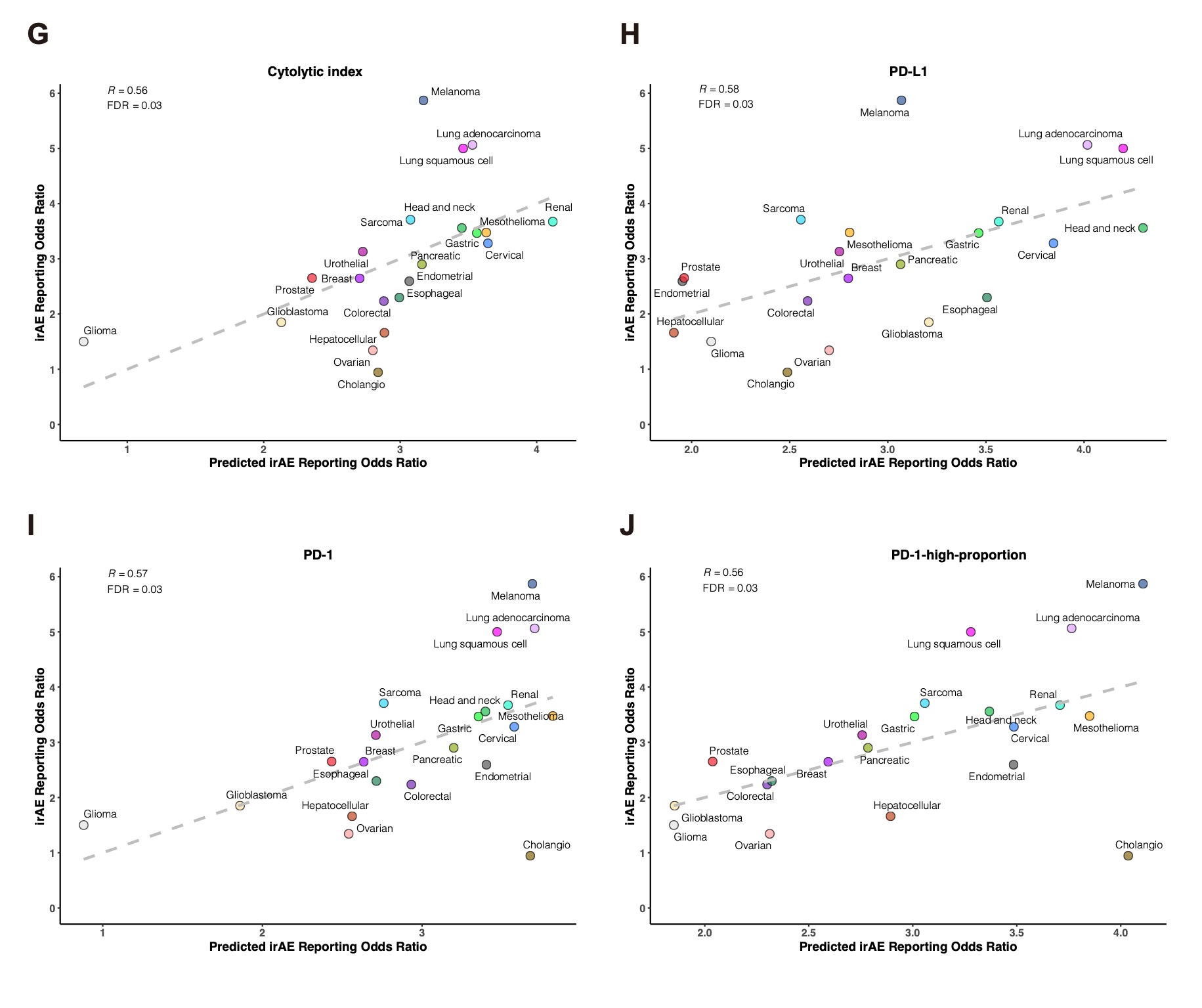
**

Cancer types are depicted in distinct colors. *R*, Pearson correlation coefficient; irAE, immune-related adverse event.

**Supplementary Figure S5. Genes Correlated With Immune-Related Adverse Event Reporting Odds Ratio During Anti-PD-1 Therapy Across Cancer Types**

**
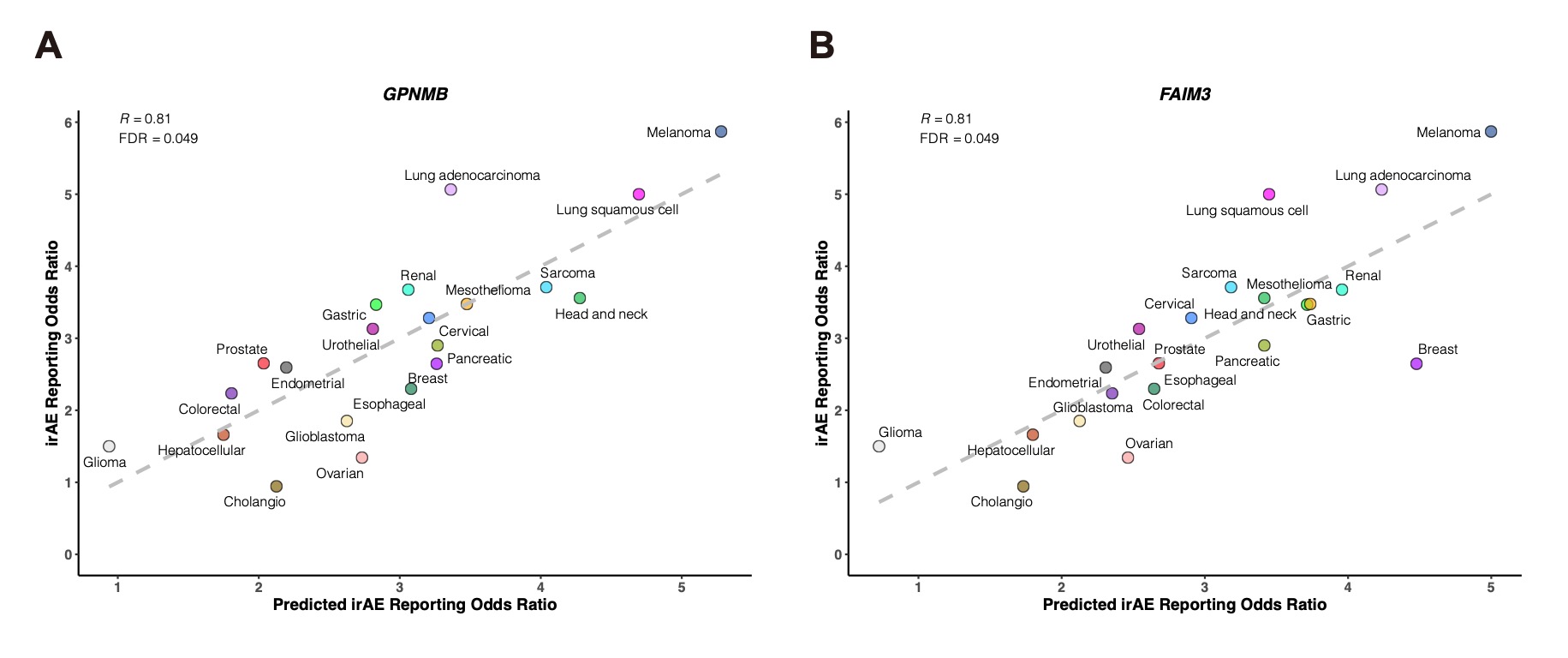
**

A and B, Correlation of *GPNMB* (A) and *FAIM3* (C) mRNA expression with the reporting odds ratio of any immune-related adverse event (irAE) across 21 cancer types which are color coded. *R*, Pearson correlation coefficient.

**Supplementary Figure S6. Protein Correlated With Immune-Related Adverse Event Reporting Odds Ratio During Anti-PD-1 Therapy Across Cancer Types**


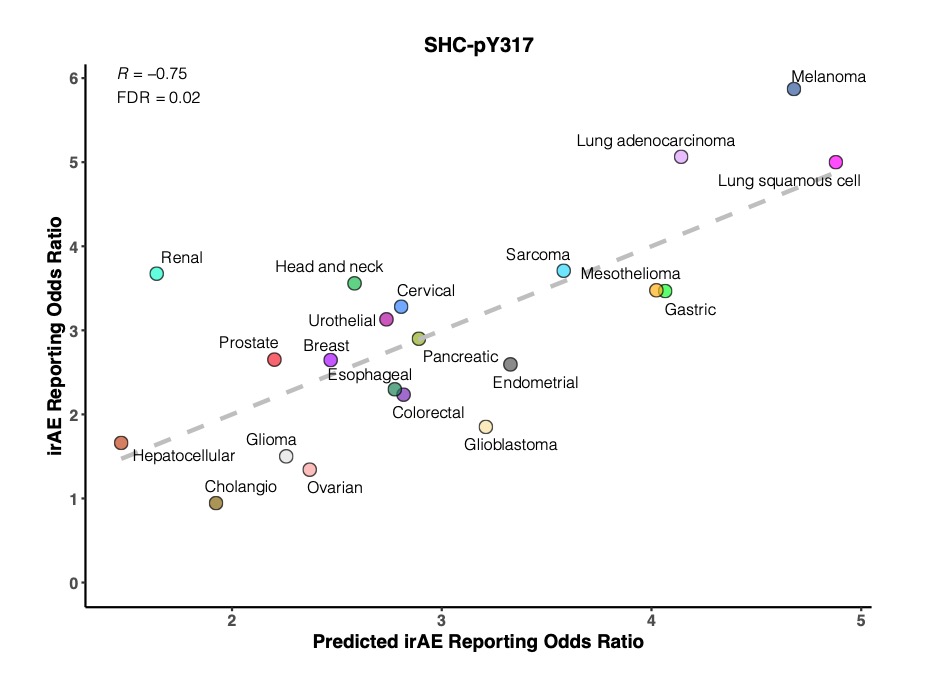


Cancer types are depicted in distinct colors. SHC-pY317, SHC phosphorylation level on Tyr317; *R*, Pearson correlation coefficient; irAE, immune-related adverse event.

**Supplementary Figure S7. MicroRNAs Correlated With Immune-Related Adverse Event Reporting Odds Ratio During Anti-PD-1 Therapy Across Cancer Types**


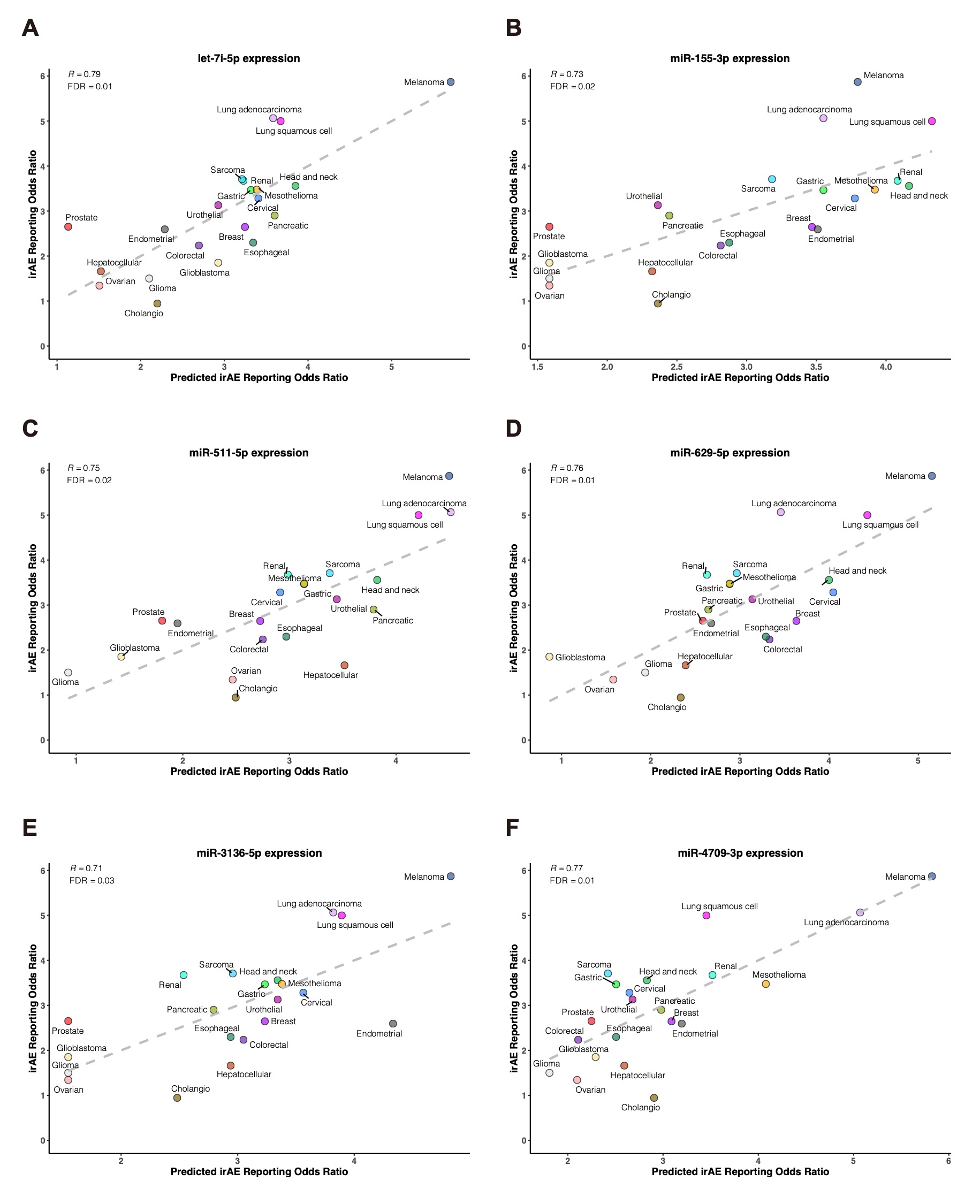


Cancer types are depicted in distinct colors. *R*, Pearson correlation coefficient; irAE, immune-related adverse event.

**Supplementary Figure S8. Performance of Bivariate Models in Predicting Immune-Related Adverse Events Risk for Combinations of Candidate Genes, Protein, and MicroRNAs**

Graph shows performance of bivariate models in predicting immune-related adverse events risk for combinations of the 11 candidate molecular factors. The Pearson correlation coefficient (*R*) is represented in colors from dark blue to dark red as shown in the color bar. Color intensity and the size of each pie is proportional to the correlation coefficient. A lack of statistical significance by log-likelihood ratio test (*P* > .05) is indicated with a gray cross.

**References**

1. Böhm R, Höcker J, Cascorbi I, Herdegen T. OpenVigil--free eyeballs on AERS pharmacovigilance data. Vol. 30, Nature biotechnology. United States; 2012. p. 137–8.

2. Bomze D, Hasan Ali O, Bate A, Flatz L. Association between immune-related adverse events during anti-PD-1 therapy and tumor mutational burden. JAMA Oncol. 2019 Aug;5(11):1633–5.

3. Goldman MJ, Craft B, Hastie M, Repečka K, McDade F, Kamath A, et al. Visualizing and interpreting cancer genomics data via the Xena platform. Vol. 38, Nature biotechnology. 2020. p. 675–8.

4. Ellrott K, Bailey MH, Saksena G, Covington KR, Kandoth C, Stewart C, et al. Scalable open science approach for mutation calling of tumor exomes using multiple genomic pipelines. Cell Syst. 2018 Mar;6(3):271-281.e7.

5. Mayakonda A, Lin D-C, Assenov Y, Plass C, Koeffler HP. Maftools: efficient and comprehensive analysis of somatic variants in cancer. Genome Res. 2018 Nov;28(11):1747–56.

6. Hoadley KA, Yau C, Hinoue T, Wolf DM, Lazar AJ, Drill E, et al. Cell-of-origin patterns dominate the molecular classification of 10,000 tumors from 33 types of cancer. Cell. 2018 Apr;173(2):291-304.e6.

7. Akbani R, Ng PKS, Werner HMJ, Shahmoradgoli M, Zhang F, Ju Z, et al. A pan-cancer proteomic perspective on The Cancer Genome Atlas. Nat Commun. 2014 May;5:3887.

8. Rooney MS, Shukla SA, Wu CJ, Getz G, Hacohen N. Molecular and genetic properties of tumors associated with local immune cytolytic activity. Cell. 2015 Jan;160(1–2):48–61.

9. Ayers M, Lunceford J, Nebozhyn M, Murphy E, Loboda A, Kaufman DR, et al. IFN-γ-related mRNA profile predicts clinical response to PD-1 blockade. J Clin Invest. 2017 Aug;127(8):2930–40.

10. McKinney EF, Lee JC, Jayne DRW, Lyons PA, Smith KGC. T-cell exhaustion, co-stimulation and clinical outcome in autoimmunity and infection. Nature. 2015 Jul;523(7562):612–6.

11. Lee JS, Ruppin E. Multiomics prediction of response rates to therapies to inhibit programmed cell death 1 and programmed cell death 1 ligand 1. JAMA Oncol. 2019 Aug;5(11):1614–8.

12. Paré L, Pascual T, Seguí E, Teixidó C, Gonzalez-Cao M, Galván P, et al. Association between PD1 mRNA and response to anti-PD1 monotherapy across multiple cancer types. Ann Oncol. 2018 Oct;29(10):2121–8.

13. Carter SL, Cibulskis K, Helman E, McKenna A, Shen H, Zack T, et al. Absolute quantification of somatic DNA alterations in human cancer. Nat Biotechnol. 2012 May;30(5):413–21.

14. Nielsen M, Andreatta M. NetMHCpan-3.0; improved prediction of binding to MHC class I molecules integrating information from multiple receptor and peptide length datasets. Genome Med. 2016 Mar;8(1):33.

15. Thorsson V, Gibbs DL, Brown SD, Wolf D, Bortone DS, Ou Yang T-H, et al. The immune landscape of cancer. Immunity. 2018 Apr;48(4):812-830.e14.

16. Aran D, Hu Z, Butte AJ. xCell: digitally portraying the tissue cellular heterogeneity landscape. Genome Biol. 2017 Nov;18(1):220.
